# Supplementary material for: An RNAi Screen Reveals an Essential Role for HIPK4 in Human Skin Epithelial Differentiation from iPSCs
Source: Stem Cell Reports. 2017 Sep 28;9(4):1234–45. doi: 10.1016/j.stemcr.2017.08.023 (PMC5639458; doi:10.1016/j.stemcr.2017.08.023)
Supplement: Document S1. Figures S1–S5 and Tables S1, S2, S4, and S5 [file mmc1.pdf]

Stem Cell Reports, Volume *9*

Supplemental Information

An RNAi Screen Reveals an Essential Role for HIPK4 in Human Skin Epithelial Differentiation from iPSCs

Lionel Larribère, Marta Galach, Daniel Novak, Karla Arévalo, Hans Christian Volz, Hans-Jürgen Stark, Petra Boukamp, Michael Boutros, and Jochen Utikal

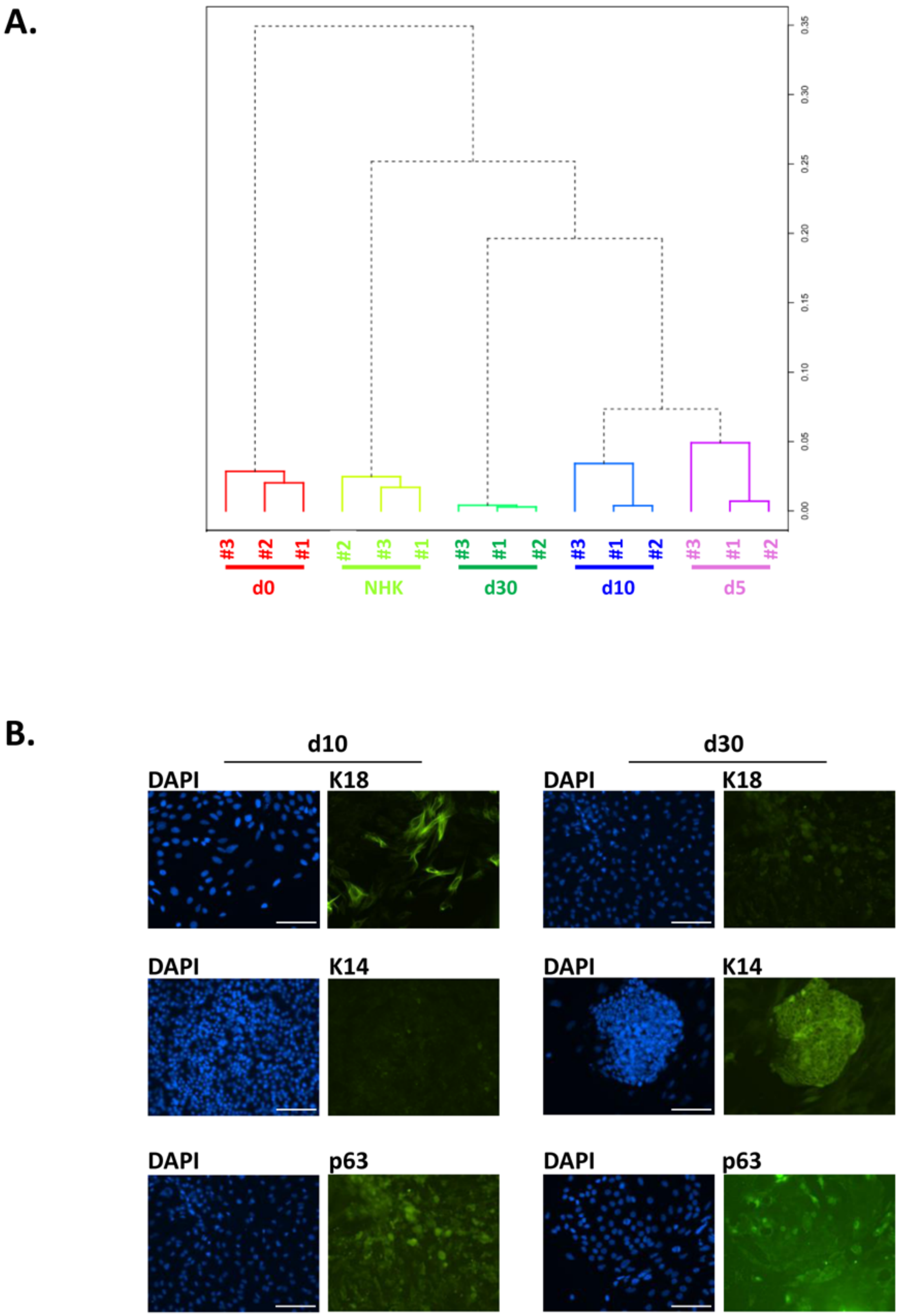

Figure S1.

A. **Related to Figure 1D:** Dendrogram of samples using normalized data with Pearson correlation and average linkage method. The branches of the tree are colored according to the groups: hiPSCs (**d0**), normal human keratinocytes (**NHK**), day 5 (**d5**), day 10 (**d10**) and day 30 (**d30**) differentiated cells. Clustering is done using the function hcluster in which the parameter correlation envokes computation of pearson type of distances.

B. **Related to Figure 1E:** Immunostaining of keratin 18 (K18), keratin 14 (K14) and p63 at day 10 and day 30 of differentiation. Nuclei were stained with DAPI. Scale bar : 100um

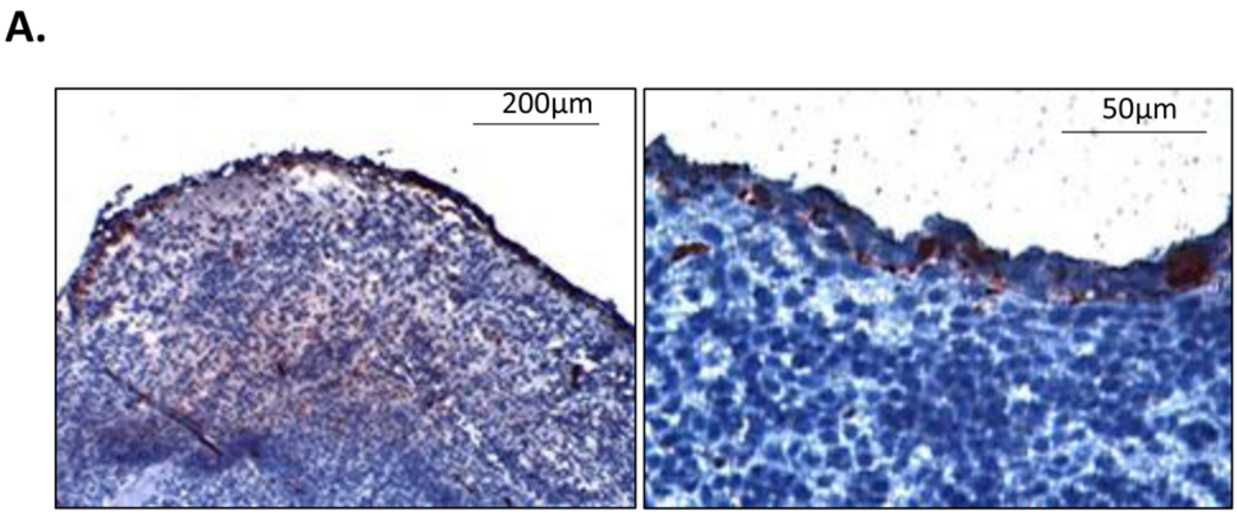

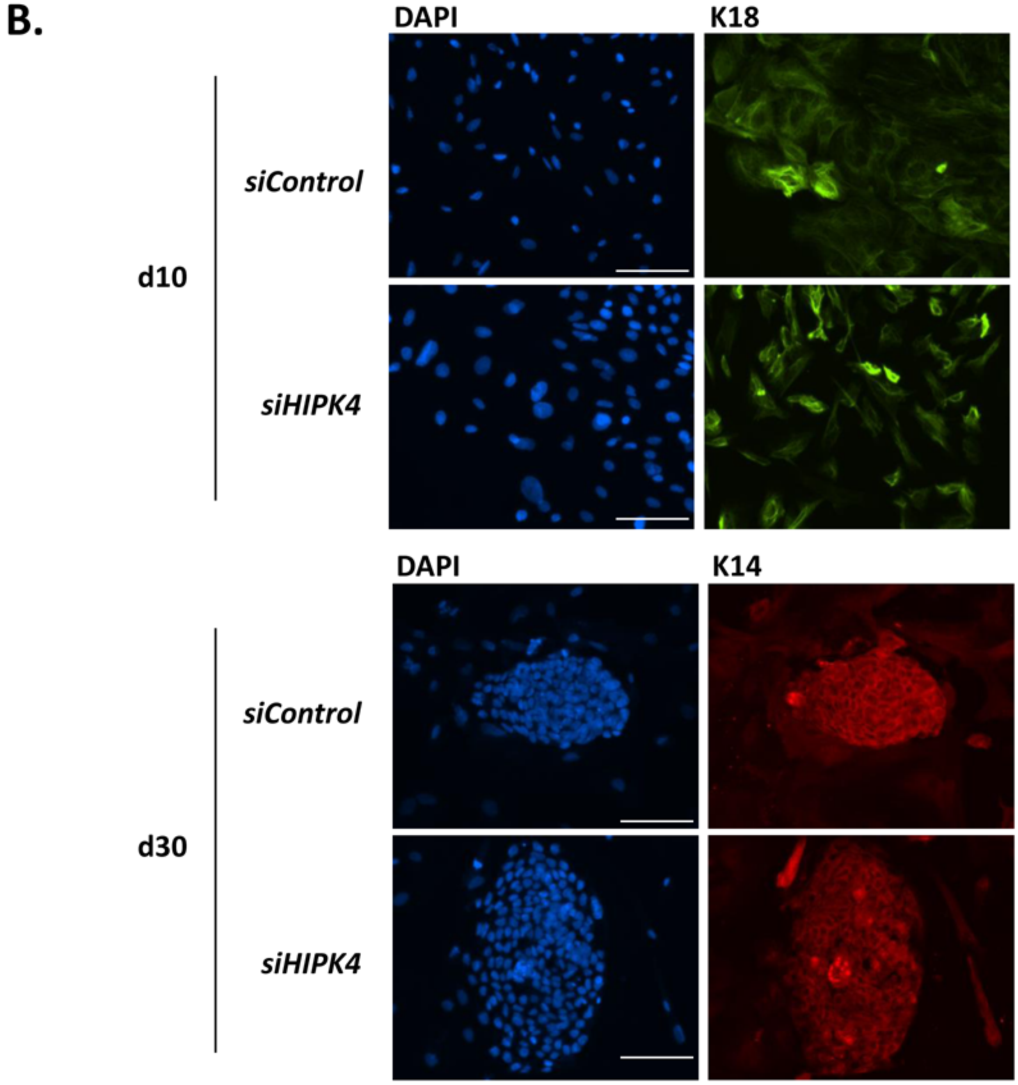

Figure S2.

A. **Related to Figure 2C:** HIPK4 in vivo expression in skin sections of mouse embryos at day E17.5 in the brain region.

B. **Related to Figure 3A:** Upregulation of K18 and K14 under HIPK4 silencing. Immunofluorescence staining against K18 at day 10 and K14 at day 30 of differentiation in control condition (*siControl*) and under HIPK4 knockdown (*siHIPK4*). Scale bar: 100 µm.

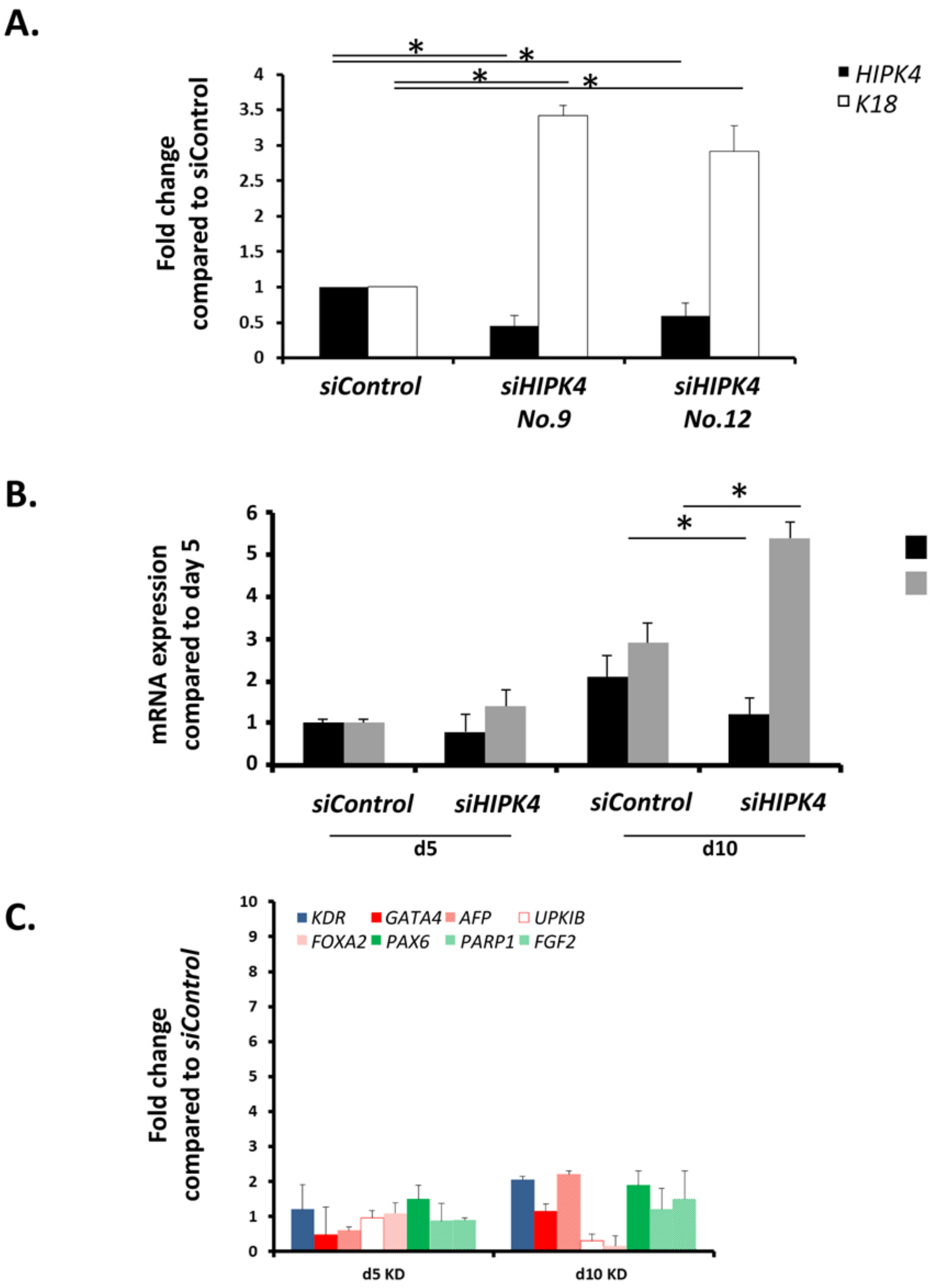

Figure S3.

A. **Related to Figure 3C: *HIPK4* silencing with single siRNAs.** qPCR analysis of *HIPK4* and Keratin 18 expression under HIPK4 knockdown with single siRNAs (*siNo.9* and *siNo.12*) or in control condition (*siControl*) at day 10 of differentiation. Data represent a mean of three independent experiments ± SEM. Statistical analysis was performed using unpaired Student's t-test (* P<0.05).

B. **Related to Figure 3C:** qPCR analysis of HIPK4 and K18 expression in a second hiPSC line (HD1) under HIPK4 silencing (*siHIPK4*) or in control condition (*siControl*) at day 5 and day 10 of differentiation. Data represent a mean of three independent experiments ± SEM. Statistical analysis was performed using unpaired Student's t-test (* P<0.1).

C. **Related to Figure 3E:** qPCR analysis of mesodermal marker (*KDR*), endodermal markers (*GATA4, AFP, UPK1B and FOXA2*) and neuroectodermal markers (*PAX6, PARP1 and FGF2*) at day 5 and day 10 under *HIPK4* knockdown. Data represent a mean of three independent experiments ± SEM.

A.

B.

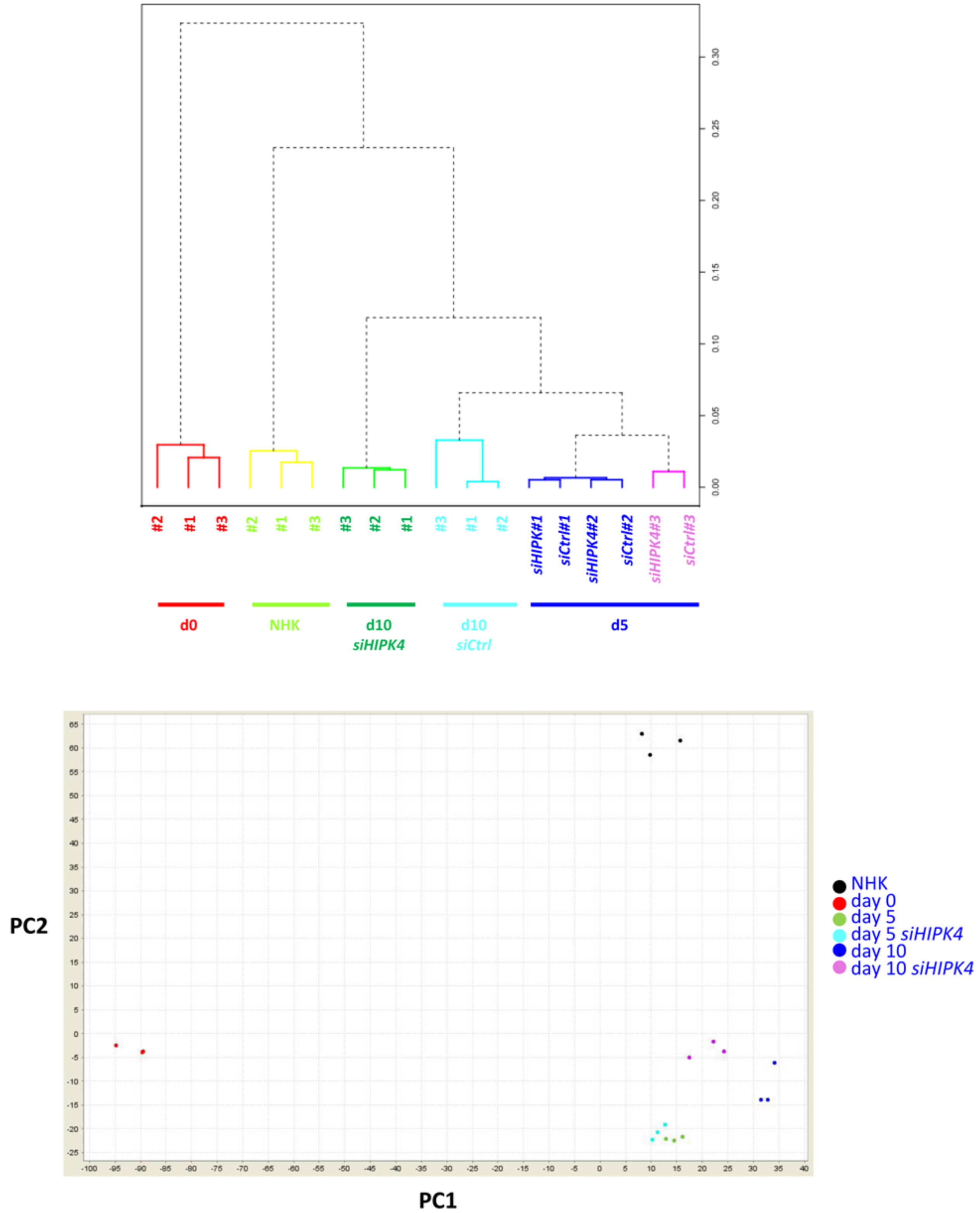

Figure S4. Related to Figure 3E: Transcriptome analysis of *HIPK4* knockdown and control samples at day 5 and day 10 of differentiation.
A. Dendrogram of samples using normalized data with Pearson correlation and average linkage method. The branches of the tree are colored according to the groups: hiPSCs (**d0**), normal human keratinocytes (**NHK**), day 5 (**d5**) and day 10 (**d10**) differentiated cells under control or HIPK4 knockdown conditions. Clustering is done using the function hcluster in which the parameter correlation envokes computation of pearson type of distances.
B. Principal component analysis of gene expression profiles on the same samples as above. Proportion of Variance PC1: 0.44 and PC2: 0.21

A.

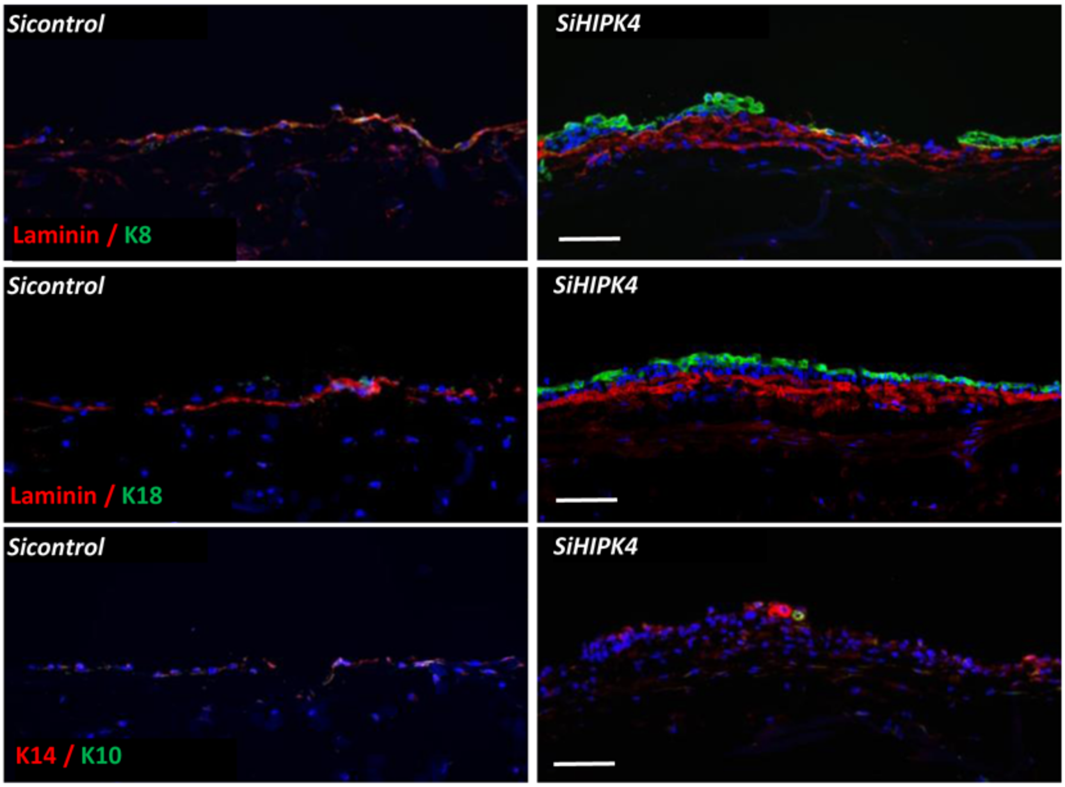

B.

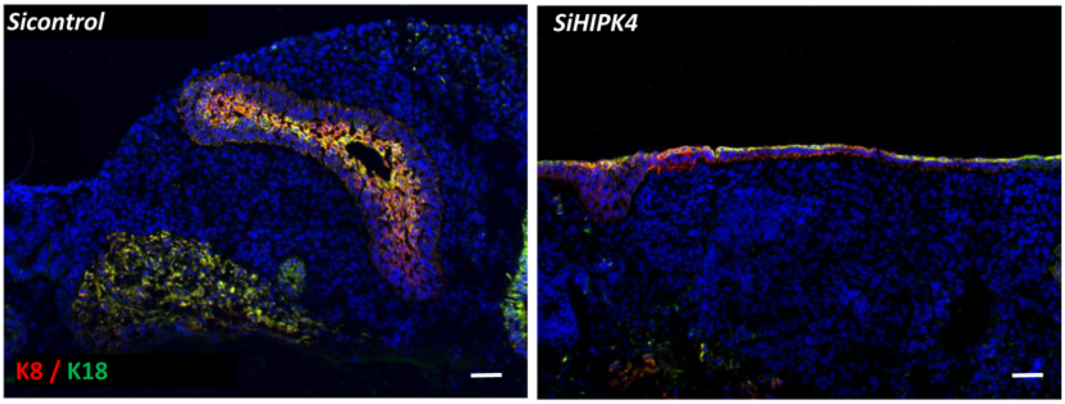

Figure S5. Related to Figure 5: *HIPK4* knockdown promotes epithelial differentiation of coculture with human dermal fibroblasts.

A. Immunostaining against Laminin b1, Keratin 8, 18, 10 and 14 in paraffin sections of 13-day old organotypic cultures (OTCs) from either *HIPK4* siRNA (*siHIPK4*) or a non-targeting siRNA (*siControl*) transfected cells. Scale bar: 100um.

B. Immunostaining against Keratin 8 and 18 in paraffin sections of 21-day old organotypic cultures (OTCs) from either HIPK4 siRNA (*siHIPK4*) or a non-targeting siRNA (*siControl*) transfected cells. OTCs of *HIPK4* siRNA treated cells show an improved directed differentiation. In contrast non-targeting siRNA treated cells demonstrate organoid structures of mixed populations of mesenchymal and epithelial cells. Scale bar: 100µm.

Table S1. Related to Figure 1B: Gene signature of endodermal, mesodermal, neuroectodermal tissue and pluripotency at day 10 of differentiation.

ENDODERM		
symbol	day 5	day 10
AFP	-1.09	-1.77
CD36	-0.04	-0.01
CDX2	0.81	0.02
CLDN6	-0.39	0.41
CTNNBL1	0.26	0.22
CXCR4	-0.01	-0.12
EOMES	-0.70	-0.76
FABP1	-0.06	-0.06
FOXA2	-0.05	-0.08
GATA4	0.04	0.03
GATA6	0.47	0.27
GDF3	-0.98	-1.01
HNF1B	-0.06	-0.11
HNF4A	0.06	0.10
MIXL1	-0.35	-0.31
PECAM1	-0.06	-0.08
SALL4	-1.88	-2.57
SOX1	-0.13	-0.07
SOX17	-0.17	-0.03
UPK1B	0.06	0.03
VEGFA	-0.31	-0.11
VEGFB	0.04	0.10
VEGFC	0.45	0.29

MESODERM		
symbol	day 5	day 10
ACVR1	0.15	-0.11
ACVR1B	0.06	0.30
ACVR1C	0.04	0.05
ACVR2A	-0.25	-0.14
ACVR2B	-0.64	-0.78
ACVR2B-AS	0.18	0.18
ACVRL1	0.13	0.03
CDH2	0.55	-0.06
CFC1	-0.07	-0.06
CFC1B	0.03	-0.01
EOMES	-0.70	-0.76
FABP4	0.12	0.28
FOXF1	0.18	-0.04
GDF3	-0.98	-1.01
GSC	0.02	-0.08
GSC2	0.05	0.19
KDR	-1.24	-1.06
MESDC1	-0.03	-0.08
MIXL1	-0.35	-0.31
NCLN	-0.67	-0.78
NOMO1	-0.55	-0.37
NOMO2	-0.17	-0.14
NOMO3	-0.21	-0.33
SNAI1	0.02	0.11
SNAI3	-0.07	-0.16
T	-0.10	-0.12
TBX6	-0.04	-0.09
TWIST2	-0.09	0.29
WNT8A	0.00	-0.12

NEUROECTODERM		
symbol	day 5	day 10
FGF1	-0.05	-0.05
FGF2	-1.59	-2.48
FGF3	-0.05	-0.10
FGFR1	-0.63	-0.78
FGFR2	-0.18	-0.11
FGFR4	0.11	0.07
PARP1	-0.56	-1.47
PAX6	0.11	-0.14

PLURIPOTENCY		
symbol	day 5	day 10
ALPL	-2.29	-3.71
DNMT3B	-1.66	-3.14
LIN28A	-0.69	-2.23
LIN28B	-0.79	-1.32
nanog	-1.96	-2.25
POU5F1	-3.50	-5.15
SOX2	-2.53	-3.61
TRIM71	-2.09	-2.46

<u>Table S2. Related to Figure 2B:</u> Screen's hits associated with epithelial differentiation

symbol	gene	Involvement (references)
BMPR1B	Bone Morphogenetic Protein Receptor, Type IB	associated with epidermis differentiation (Botchkarev et al., 1999; Panchision & Pickel, 2001)
IKBKAP	Inhibitor Of Kappa Light Polypeptide Gene Enhancer In B-Cells, Kinase Complex-Associated Protein	promote epidermal differentiation (Hu et al., 2001)
IKBKE	Inhibitor of kappa light polypeptide gene enhancer in B-cells, kinase epsilon	
MAPK1	Mitogen-Activated Protein Kinase 1	viability and proliferation of keratinocyte (Scholl et al., 2007)
MAP2K1	Mitogen-Activated Protein Kinase Kinase 1	
MAP2K2	Mitogen-Activated Protein Kinase Kinase 2	
MAP2K4	Mitogen-Activated Protein Kinase Kinase 4	
MAP3K3	Mitogen-Activated Protein Kinase Kinase Kinase 3	
MAP3K12	Mitogen-Activated Protein Kinase Kinase Kinase 12	

Table S4. Related to Figure 2B: Screen's top candidate gene list

	GeneID	z-score
promoter candidates	STK33	4.04
	TEK	3.77
	MYLK	3.43
	GSK3B	3.38
	CDKN2B	3.3
	GCK	2.98
	MAP2K4	2.86
	MAPKAPK3	2.78
	PAK1	2.75
	CDKL1	2.72
	MYO3A	2.65
	SRPK2	2.64
	GTF2H1	2.62
	CDK11B,CDK11A	2.52
	MAP2K2	2.43
	MAP2K3	2.41
	TYK2	2.38
	STYK1	2.3
	RBKS	2.29
	CDK7	2.25
	GRK7	2.25
	**ADCK2**	**2.24**
	ADRB2	2.2
	ZAP70	2.18
	PHKA2	2.17
	CDKN3	2.16
	MVK	2.1
	TRAT1	2.06
	WNK4	2.04
	GK2	2.02
	GMFB	2.02
	XYLB	2
	IPPK	1.99
	JAK1	1.99
	CAMK2B	1.98
	PIP5K1A	1.97
	SOCS5	1.96
	DUSP10	1.92
	CDK5R1	1.9
	CSNK2B	1.89
	MAPK1	1.85
	SHPK	1.8
	IRAK3	1.77
	DGKQ	1.72
	DYRK2	1.72
	SCYL2	1.71
	NEK9	1.7

	GeneID	z-score
inhibitor candidates	CSNK1E	-1.55
	DBF4	-1.56
	ACVR2A	-1.57
	YWHAQ	-1.57
	CINP	-1.59
	MLKL	-1.59
	AATK	-1.6
	TRIB3	-1.61
	AAK1	-1.62
	PRKD1	-1.64
	**HIPK3**	**-1.66**
	EIF2AK3	-1.69
	EPHA3	-1.69
	FASTK	-1.7
	GUCY2F	-1.71
	POM121L10P,BCR	-1.71
	FGFR1	-1.72
	SRPK1	-1.72
	BMP2K	-1.73
	CHEK2	-1.73
	CHKA	-1.73
	EPHA4	-1.73
	EIF2AK1	-1.74
	TEX14	-1.74
	CHKB	-1.75
	BLK	-1.77
	**HIPK4**	**-1.77**
	TTBK2	-1.79
	TAF1L,TAF1	-1.83
	TPK1	-2.01
	SLK	-2.1
	TSSK1B,DGCR14,TSSK2	-2.14
	UCK2	-2.21
	TTK	-2.38
	PBK	-2.42
	TXK	-3.01

	CKS1B	1.69
	PAK2	1.69
	CARD10	1.68
	MST4	1.68
	TWF2	1.65
	AVPR1A	1.62
	CD4	1.59
	ILKAP	1.56
	TLR1	1.56
	ARAF	1.54
	CDK10	1.54
	PRKDC	1.52
	PRKCQ	1.51
	CD7	1.5
	ZAK	1.5

Table S5. Related to Figure 3D: Gene signature of endodermal, mesodermal, neuroectodermal tissue and of pluripotency.

ENDODERM			MESODERM			NEUROECTODERM		
**symbol**	**day 5KD**	**day 10KD**	**symbol**	**day 5KD**	**day 10KD**	**symbol**	**day 5KD**	**day 10KD**
KRT19	0.02	-1.93	HAND1	-0.25	-3.08	FGF1	0.01	0.01
CLDN6	-0.11	-1.80	WNT3A	0.27	-0.45	FGF3	-0.03	0.11
SOX7	0.00	-1.59	NOMO1	0.15	-0.44	FGFR1	-0.08	0.13
CDH5	0.13	-1.54	ACVR1B	0.08	-0.40	FGFR2	0.03	-0.17
CTNNBL1	-0.05	-1.13	TWIST1	0.02	-0.40	FGFR3	-0.13	-0.91
KRT7	0.04	-0.99	NCLN	0.14	-0.37	FGFR4	0.01	-0.06
CD14	-0.02	-0.51	GDF3	-0.02	-0.23	PARP1	0.04	-0.08
SALL4	0.07	-0.47	TWIST2	0.08	-0.22	PAX6	-0.02	0.54
FOXA1	-0.14	-0.46	GSC2	0.02	-0.18	**PLURIPOTENCY**		
GDF3	-0.02	-0.23	KDR	0.03	-0.17	**symbol**	**day 5KD**	**day 10KD**
SOX17	0.14	-0.17	ACVR2B-AS	0.01	-0.17	LIN28A	0.03	-2.87
GATA6	0.04	-0.14	SNAI1	0.01	-0.09	DNMT3B	-0.01	-1.55
CDX2	0.03	-0.10	SNAI2	0.02	-0.08	KLF4	0.06	-0.93
SOX1	0.01	-0.05	FOXF1	-0.09	-0.02	ABCG2	-0.21	-0.84
CXCR4	0.05	-0.04	MIXL1	0.17	-0.02	ACVR1B	-0.33	-0.81
HNF4A	-0.06	-0.02	SNAI3	-0.05	-0.01	TRIM71	0.17	-0.72
MIXL1	0.17	-0.02	GSC	-0.11	-0.01	ALPL	0.15	-0.70
FABP1	-0.03	0.05	NOMO2	0.02	-0.01	POU5F1	0.07	-0.42
GATA4	0.08	0.06	CFC1	0.03	0.02	TPBG	0.02	-0.15
VEGFA	0.06	0.07	CFC1B	0.04	0.02	LEFTY1	-0.05	-0.10
FOXA2	-0.01	0.07	TBX6	-0.10	0.07	ACVR2B	-0.05	-0.06
CD36	-0.07	0.15	T	0.06	0.07	FUT2	0.04	-0.04
PECAM1	-0.02	0.17	ACVR1C	0.02	0.07	LEFTY2	0.08	0.02
AFP	-0.27	0.17	ACVR2B	-0.07	0.10	CFC1	0.03	0.02
HNF1B	0.03	0.18	WNT8A	-0.02	0.10	TERT	0.11	0.04
UPK1B	0.06	0.28	MESDC1	-0.03	0.11	LIN28B	-0.03	0.08
EOMES	0.03	0.35	ACVRL1	-0.21	0.15	SOX2	0.03	0.54
VEGFB	-0.05	0.47	ACVR2A	0.08	0.22	KLF5	0.05	0.61
			EOMES	0.03	0.35			
			NOMO3	-0.05	0.43			
			ACVR1	0.02	0.74			
