# Supplementary material for: An RNAi Screen Reveals an Essential Role for HIPK4 in Human Skin Epithelial Differentiation from iPSCs
Source: Stem Cell Reports. 2017 Sep 28;9(4):1234–45. doi: 10.1016/j.stemcr.2017.08.023 (PMC5639458; doi:10.1016/j.stemcr.2017.08.023)
Supplement: Document S2. Article plus Supplemental Information [file mmc4.pdf]

Stem Cell Reports
Article

ISSCR

OPEN ACCESS

An RNAi Screen Reveals an Essential Role for HIPK4 in Human Skin Epithelial Differentiation from iPSCs

Lionel Larribère,[1,2,8,*] Marta Galach,[1,2,8] Daniel Novak,[1,2] Karla Arévalo,[1,2] Hans Christian Volz,[3,4,5] Hans-Jürgen Stark,[6] Petra Boukamp,[6,7] Michael Boutros,[3,4] and Jochen Utikal[1,2,*]

[1]Skin Cancer Unit (G300), German Cancer Research Center (DKFZ), Im Neuenheimer Feld 280, 69121 Heidelberg, Germany
[2]Department of Dermatology, Venereology and Allergology, University Medical Center Mannheim, Heidelberg University, 68167 Mannheim, Germany
[3]Division of Signaling and Functional Genomics, German Cancer Research Center (DKFZ)
[4]Department of Cell and Molecular Biology, Heidelberg University
69120 Heidelberg, Germany
[5]Department of Cardiology, Heidelberg University, 69120 Heidelberg, Germany
[6]Genetics of Skin Carcinogenesis, German Cancer Research Center (DKFZ), 69120 Heidelberg, Germany
[7]IUF–Leibniz Research Institute for Environmental Medicine, 40021 Düsseldorf, Germany
[8]Co-first author
*Correspondence: l.larribere@dkfz.de (L.L.), j.utikal@dkfz.de (J.U.)
http://dx.doi.org/10.1016/j.stemcr.2017.08.023

SUMMARY

Molecular mechanisms responsible for the development of human skin epithelial cells are incompletely understood. As a consequence, the efficiency to establish a pure skin epithelial cell population from human induced pluripotent stem cells (hiPSCs) remains poor. Using an approach including RNAi and high-throughput imaging of early epithelial cells, we identified candidate kinases involved in their differentiation from hiPSCs. Among these, we found HIPK4 to be an important inhibitor of this process. Indeed, its silencing increased the amount of generated skin epithelial precursors at an early time point, increased the amount of generated keratinocytes at a later time point, and improved growth and differentiation of organotypic cultures, allowing for the formation of a denser basal layer and stratification with the expression of several keratins. Our data bring substantial input regarding regulation of human skin epithelial differentiation and for improving differentiation protocols from pluripotent stem cells.

INTRODUCTION

Lineage-specific differentiation protocols from either human embryonic stem cells (hESCs) or human induced pluripotent stem cells (hiPSCs) toward the keratinocyte lineage represent a useful tool to generate human skin tissue (Green et al., 2003; Guenou et al., 2009; Itoh et al., 2011; Iuchi et al., 2006; Metallo et al., 2008; Tolar et al., 2011). Several applications can be found for these protocols, such as investigating the molecular mechanisms involved during the development of this cell lineage or *in vitro* modeling of skin diseases. Indeed, many iPSC-based models have already been reported for several tissues including brain, heart, blood, and skin (Carvajal-Vergara et al., 2010; Galach and Utikal, 2011; Kondo et al., 2013; Larribere et al., 2015; Nissan et al., 2011; Raya et al., 2009).

Current protocols on skin epithelial differentiation require the use of specific culture conditions as well as different coating or supportive feeder layers. They also need to be reproducible, robust, and efficient enough to deliver high amounts of pure differentiated cells, especially if the future goal is a clinical application. In addition, these protocols should be validated by functional assays, for example, grafting hiPSC-generated keratinocytes on organotypic cultures to prove their ability to build a whole stratified epithelium (Aberdam, 2004; Coraux et al., 2003; Guenou et al., 2009; Itoh et al., 2011). Nevertheless, the efficiency to establish not only a pure keratinocyte population from PSCs but also skin epithelial precursors remains poor to date, one of the reasons being the lack of knowledge of the molecular mechanisms regulating the early steps of the epithelial/ectodermal commitment.

Large-scale loss-of-function (RNAi) screening offers a systematic genetic approach to study lineage-specific differentiation mechanisms. To identify key actors of the epithelial/ectodermal commitment, we performed a kinome RNAi screen targeting a total of 719 kinases, followed by differentiation lasting 10 days (Erfle et al., 2007). We then used a live-cell assay and automatic microscopy based on keratin 18 (K18) expression to monitor the amount of generated epithelial cells. We identified 62 activators and 36 inhibitors of this differentiation, among which the homeodomain interacting protein kinase 4 (HIPK4) was validated as a novel negative regulator.

RESULTS

Characterization of Human Skin Epithelial Cells

hiPSCs were generated in our laboratory from fibroblasts of healthy donors (Larribere et al., 2015) and were differentiated for 5 days, 10 days, and 30 days toward the skin epithelial lineage by maintaining them in defined keratinocyte serum-free medium (DKSFM) containing retinoic acid

This is an open access article under the CC BY license (http://creativecommons.org/licenses/by/4.0/).

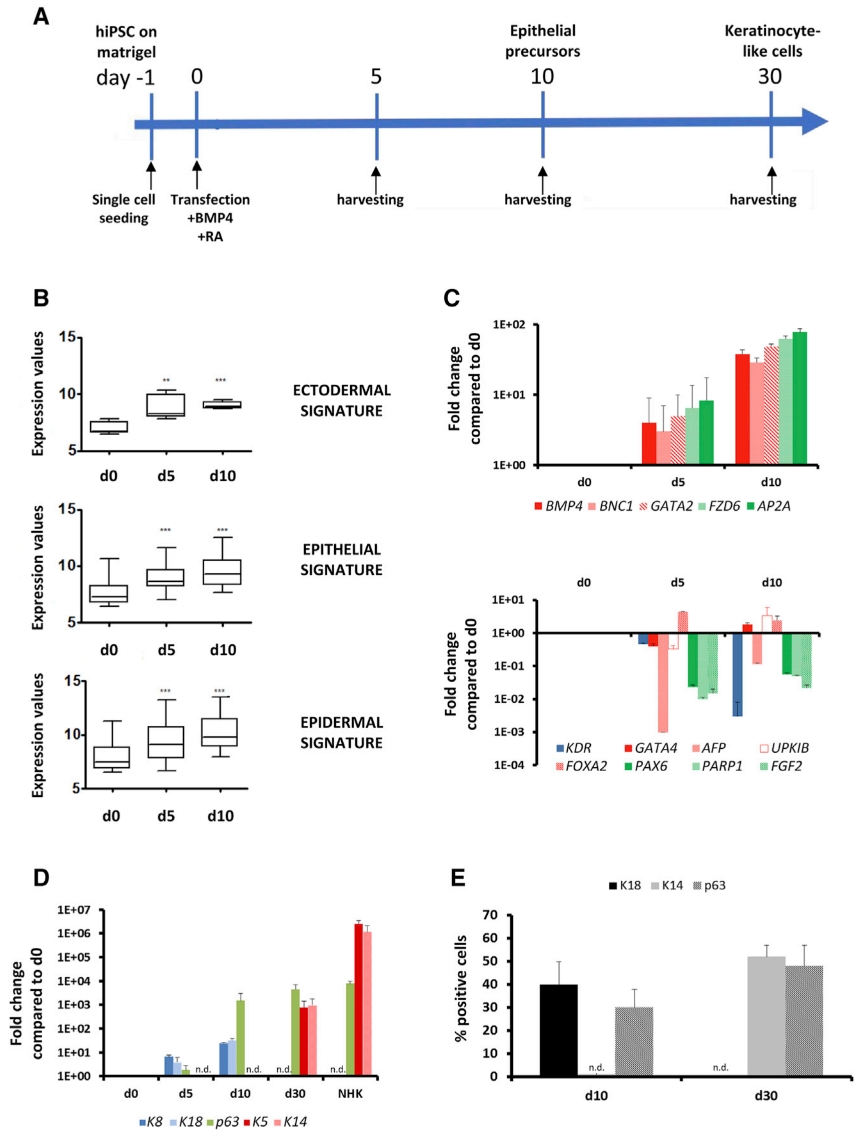

Figure 1. Characterization of Human Skin Epithelial Cells

(A) Schematic of the skin epithelial differentiation protocol.

(B) Microarray-based $\log_2$-expression of known gene signatures associated with ectodermal development, and epithelial and epidermal differentiation at day 0, day 5, and day 10 of differentiation. Statistical analysis was performed using a two-tailed paired Student's t test (**$p < 0.001$, ***$p < 0.0001$).

(C) Top panel: qPCR analysis of *BMP4* (ectodermal/epithelial marker), *BNC1* (ectodermal/epidermal marker), *GATA2* (ectodermal marker), *FZD6*, and *AP2A* (epithelial markers), at day 0, day 5, and day 10 of differentiation. Bottom panel: qPCR analysis of mesodermal marker (*KDR*), endodermal markers (*GATA4*, *AFP*, *UPK1B*, and *FOXA2*), and neuroectodermal markers (*PAX6*, *PARP1*, and *FGF2*).

(D) qPCR analysis of keratin 8 (*K8*), keratin 18 (*K18*), keratin 5 (*K5*), keratin 14 (*K14*), and *p63* expression at day 0, day 5, day 10, and day 30 of differentiation. Normal human keratinocytes (NHK) were used as control. n.d., not detectable.

(E) Immunofluorescence staining against K18, K14 and p63 at day 10 and day 30 of differentiation. Histogram represents the percentage of positive cells.

Data in (B) to (E) represent a mean of three independent experiments ± SEM.

(RA) (1 μM) and bone morphogenetic protein 4 (BMP4) (10 ng/mL), according to the protocol of Itoh et al. (2011) (Figure 1A). Gene expression profiling of the differentiated cells at day 5 and day 10 showed an increased expression of ectodermal, epithelial, and epidermal gene signatures compared with day 0 (hiPSCs) (Figure 1B). In particular, day-10 differentiated cells showed increased expression of several collagens, integrins, keratins, and laminins. Moreover, the pluripotency gene signature (including *SOX2*, *OCT4*, and *NANOG*) was downregulated and gene signatures of undesired lineages such as neuroectoderm, endoderm, or mesoderm were either not expressed or even downregulated compared with day 0 (Table S1). These data were then confirmed by qPCR for some of the ectodermal/epithelial markers: *BMP4*, *BNC1*, *GATA2*, *FZD6*, and *AP2A* were all upregulated at day 5 and day 10 compared with day 0 (Figure 1C, top panel). Conversely, mesodermal marker (*KDR*), endodermal markers (*GATA4*, *AFP*, *UPK1B*, and *FOXA2*) and neuroectodermal markers (*PAX6*, *PARP1*, and *FGF2*) were expressed at very low levels compared with day 0 (Figure 1C, bottom panel).

Furthermore, the differentiation at day 30 showed a cell phenotype and a gene expression profile closely resembling that of normal human keratinocytes (NHK). Indeed, a hierarchical clustering representation of the samples showed that cells differentiated at day 5, day 10, and day 30 presented closer similarities to NHK than at day 0 (Figure S1A). This suggests that cells were already committed to an epithelial identity as early as day 5 of differentiation. Additionally, we confirmed by qPCR at day 5 and day 10 an

increased expression of epithelial markers such as keratin 8 (*K8*) and *K18*, expression of which then decreased at day 30 and switched to an increased expression of keratinocyte markers such as keratin 5 (*K5*) and keratin 14 (*K14*). The expression of *p63*, a transcription factor involved in epidermal proliferation and stratification, was upregulated at day 10 and increased at day 30 (Figure 1D).

Finally, immunostaining of K18 at day 10 (subsequently used to monitor the RNAi screen, see below) presented 40% positive cells compared with 0% in the undifferentiated control condition (Figures 1E and S1B). As expected, day-10 differentiated cells were also positive for p63 staining (30%) but not for K14, since this marker's expression increases later during the differentiation. Indeed, day-30 differentiated cells were positive for K14 (50%) and p63 (50%) staining but no longer for K18 due to its early transient expression.

Together, these data show that our differentiation protocol generates day-5 and day-10 differentiated cells with an ectodermal/skin epithelial identity and generates day 30 differentiated cells presenting a keratinocyte-like phenotype and identity. In the following experiments, we focused on day-10 differentiated cells to investigate the molecular mechanisms that regulate early skin epithelial differentiation.

High-Throughput RNAi Screen Analysis Identifies HIPK4 as an Inhibitor of Skin Epithelial Differentiation

We next performed an RNAi-based screen during early epithelial differentiation to identify key regulators, with the ultimate goal of improving its low efficiency. In brief, hiPSCs were single-cell seeded on Matrigel-coated 384-well plates and transfected directly in the differentiation medium with a kinome RNAi library containing small interfering RNAs (siRNAs) targeting kinases and kinase-regulatory proteins. After 10 days, the cells were stained against K18 and subsequently image processed with an automated microscope (Figure 2A).

During the screen's image analysis, the number of $K18^{+}$ cells was determined, the values normalized per assay plate, and the *Z* scores calculated. Figure 2B shows the distribution of the control groups by their *Z* score and indicates a clear distinction between the positive control, in which conditions were applied to promote differentiation (cultured with DKSFM containing RA + BMP4) and the negative control, in which conditions were used to maintain pluripotency (cultured with mTeSR medium for 10 days). Two independent screens were performed, and only the results that increased or reduced the percentage of $K18^{+}$ cells from the sample median by at least two median absolute deviations were considered as hits. A gene was considered as a potential regulator of epithelial cells when both replicates were scored as hits.

Out of 232 hits, 35 genes were involved in tissue development functions (such as the fibroblast growth factor receptor [*FGFR*] family) as expected for this assay based on stem cells' early differentiation. We also found *BMPR1B* (bone morphogenetic protein receptor, type IB), a member of the BMP pathway, which supports our epithelial differentiation. BMP targets are indeed known to influence the development, differentiation, and proliferation of the epidermis (Metallo et al., 2008), and *BMPR1B* was described to be associated with epidermal differentiation (Botchkarev et al., 1999; Panchision and Pickel, 2001). In addition, we found genes associated with IKKs (inhibitor of kappa light polypeptide gene enhancer in B cells), such as *IKBKAP* (inhibitor of kappa light polypeptide gene enhancer in B cells, kinase complex-associated protein), which is a regulator of IKKs, and *IKBKE* (inhibitor of kappa light polypeptide gene enhancer in B cells, kinase epsilon) which is a non-canonical IKK. Of note, IKKα can promote epidermal differentiation independently of its nuclear factor κB (NF-κB) function (Hu et al., 2001). Finally, members of the MAPK pathway were also involved in K18 regulation: *MAPK1* (mitogen-activated protein kinase 1), *MAP2K1* to *MAP2K5* (mitogen-activated protein kinase kinase 1–5), *MAP3K3* and *MAP3K12* (mitogen-activated protein kinase kinase kinase 3 and 12). Together, these data support the functional relevance of our screen's hit list in the regulation of epithelial differentiation (Tables S2 and S3).

Next, genes with a *Z* score lower than the averaged positive controls were identified as inhibitor candidate genes and genes with a higher *Z* score than the averaged negative controls were identified as promoter candidate genes (Table S4). One of the promoter candidate genes was *ADCK2* (AarF domain containing kinase 2), mutation of which is associated with Klippel-Feil syndrome, a disease involving segmentation defects during early development. This gene was used as a representative example of differentiation promoter gene as the $K18^{+}$ cell number remained close to 1% after a differentiation of 10 days (Figure 2C). On the other hand, one inhibitor candidate gene, *HIPK4*, encodes for a conserved serine/threonine kinase belonging to the HIPK family (homeodomain interacting protein kinase 1–4), which plays a role in a large set of cell functions, including differentiation (He et al., 2010). *HIPK4* is described to be expressed in human skin according to the GeneCards online database (https://genecards.weizmann.ac.il/v3/cgi-bin/carddisp.pl?gene=HIPK4#expression). Moreover, we observed Hipk4 expression in the skin of mouse embryos, suggesting a potential role for this protein in the early development of the skin (Figure S2A).

Indeed, quantification of $K18^{+}$ cells in *HIPK4* knockdown condition after 10 days of differentiation indicated 85%

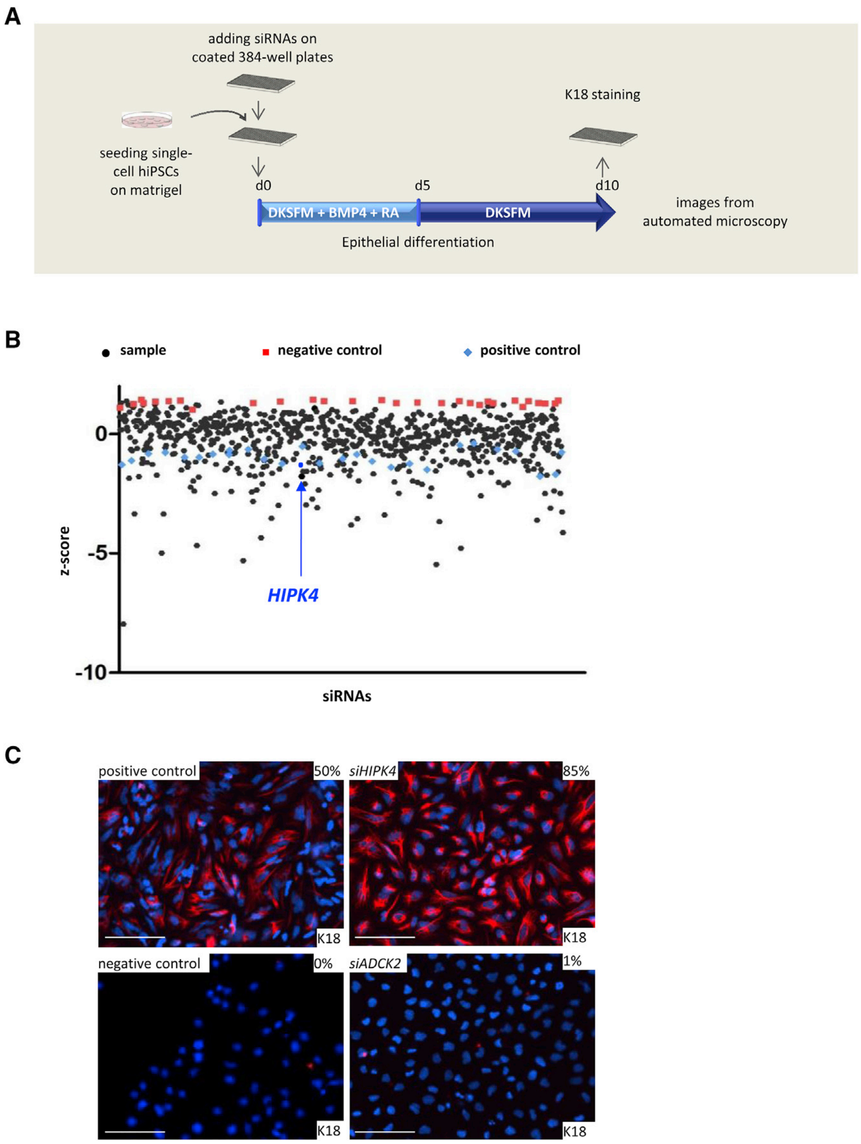

Figure 2. High-Throughput RNAi Screen Identifies HIPK4 as an Inhibitor of Skin Epithelial Differentiation

(A) Schematic of the RNAi screen workflow. Single hiPSCs were reverse transfected with a kinome RNAi library and differentiated into epithelial precursors. At day 10, immunofluorescence staining against K18 was performed and cells were imaged by automated microscopy.

(B) Scatterplot of data where each siRNA from the screen at day 10 is represented as the *Z*-score value. Plot includes negative (red, *Z* score = +1.3) and positive (blue, *Z* score = −1) controls. Positive control condition corresponds to cells transfected with *siControl* and differentiated for 10 days. Negative control condition corresponds to cells maintained in pluripotency condition for 10 days. Arrow marks siRNA against *HIPK4* as a sample with increased numbers of K18$^+$ cells compared with the positive control.

(C) Representative images of K18 immunostaining and quantification of percentage of positive cells after 10 days of differentiation with or without *siHIPK4* or *siADCK2*. Scale bar, 100 μm.

positive cells compared with 50% in the positive control condition (*siControl*). As expected, no K18 expression was found in the negative control condition (cells maintained in pluripotency condition) (Figure 2C). Overall, this RNAi screen allowed us to identify several putative regulators of human skin epithelial differentiation, and specifically suggests HIPK4 as a strong inhibitor of this process.

Validation of HIPK4 as an Inhibitor of Skin Epithelial Differentiation

In the next step, we quantified by immunostaining the number of cells positive for K18, K14, and p63 at day 10 and day 30 of differentiation (Figures 3A and S2B). At day 10, *HIPK4* silencing induced an increase of K18$^+$ cells (80%) compared with the *siControl* condition (40%). As expected at this time point, no K14$^+$ cells were observed. Similarly at day 30, *HIPK4* silencing induced a small but significant increase of K14$^+$ cells (65%) compared with the *siControl* condition (50%). Interestingly, at day 10, the number of p63$^+$ cells was not affected by *HIPK4* knockdown (around 30%) but increased significantly at day 30 compared with the *siControl* condition (from 50% to 65%). These data suggest that the inhibitory role of HIPK4 on K18 expression before day 10 is independent of p63. At a later time point, however (day 30), the increase in K14$^+$ and p63$^+$ cells in the *siHIPK4* condition may simply be due to an increased pool of day-10 precursors that matured in keratinocytes.

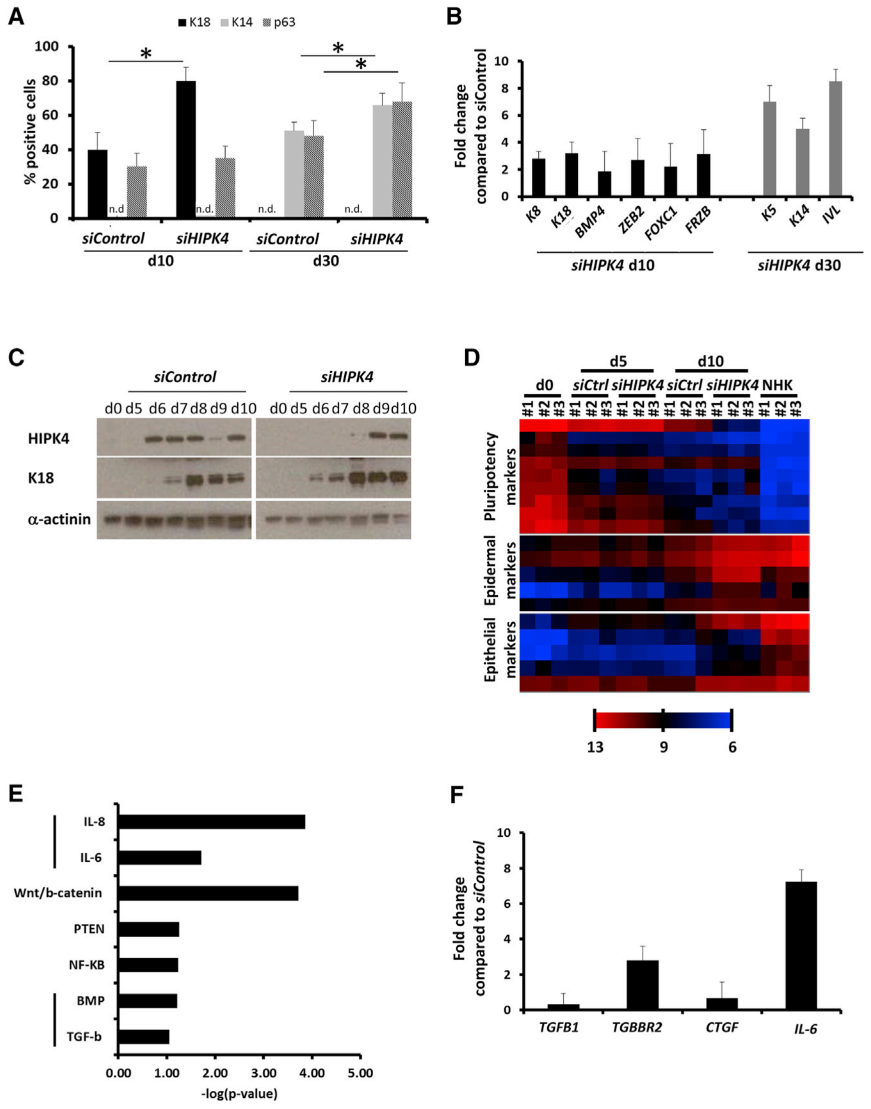

Figure 3. Validation of HIPK4 as an Inhibitor of Skin Epithelial Differentiation

(A) Immunofluorescence staining against K18, K14, and p63 at day 10 and day 30 of differentiation in control condition and under *HIPK4* knockdown. Histogram represents the percentage of positive cells. Data represent a mean of three independent experiments ± SEM. Statistical analysis was performed using unpaired Student's t test (*$p < 0.05$). n.d., not detected.

(B) qPCR analysis of epithelial markers under *HIPK4* knockdown at day 10 (black bars) and keratinocyte makers under *HIPK4* knockdown at day 30 (gray bars). Values represent fold changes compared with the respective controls and are mean of three independent experiments ± SEM.

(C) Western blot analysis of HIPK4 and K18 expression in the indicated conditions.

(D) Heatmap representing a hierarchical gene clustering of pluripotency, epidermal, and epithelial markers generated from hiPSCs (d0), normal human keratinocytes (NHK), and day 5 (d5) and day 10 (d10) of differentiation with or without *HIPK4* knockdown. Color code represents $\log_2$-expression values.

(E) Ingenuity Pathway Analysis showing some of the top regulated signaling pathways after *HIPK4* knockdown at day 10 of differentiation (compared with *siControl*).

(F) qPCR analysis of *IL-6* and three members of the TGF-β signaling (*TGFB1*, *TGFBR2*, and *CTGF*) under *HIPK4* knockdown at day 10. Data represent a mean of three independent experiments ± SEM.

We also analyzed the expression of epithelial markers at day 10 or keratinocyte markers at day 30 in these samples. In addition to K8 and K18, the expression of *BMP4*, *ZEB2*, *FOXC1*, and *FRZB* was upregulated by at least 2-fold at day 10 under *HIPK4* knockdown compared with the *siControl* condition. Similarly, the expression of *K5*, *K14*, and involucrin (*IVL*) was upregulated by at least 5-fold at day 30 in the *HIPK4* knockdown condition (Figure 3B). The regulation of K18 expression by HIPK4 was also analyzed by western blot in a kinetic experiment from day 5 to day 10 (Figure 3C). HIPK4 expression, which starts at day 6 in the control condition, was delayed to day 9 under *HIPK4* silencing. The restoration of HIPK4 expression after day 9 in the knockdown condition was probably due to the combined effects of transient siRNA silencing and increased expression during differentiation. Consequently, K18 expression, which started at day 7 in the control condition, was already expressed at day 6 in the *HIPK4* knockdown condition, and accumulated at day 10 to higher levels than in the control condition.

Because the screen's potential off-target effects are to be expected, the differentiation experiment was repeated with two independent *HIPK4* siRNAs. The analysis at day 10 revealed that each single *HIPK4*-specific siRNA led to gene silencing (50% and 40%, respectively). Consistently, the increase in K18 expression under *HIPK4* silencing was still observed (Figure S3A). Moreover, the negative regulation of HIPK4 on K18 expression was reproduced in a second hiPSC line (HD1). At day 5, transfection with *siHIPK4* did not significantly increase K18 expression, likely due to the absence of *HIPK4* at this time point; however, at day 10 *siHIPK4* caused more than a 50% reduction in *HIPK4* expression and a 2-fold increase of K18 expression compared with *siControl* (Figure S3B).

For better characterization of the differentiated cell populations, microarray analysis of transcriptome profiles from control and *HIPK4* knockdown samples at day 5 and day 10 was conducted together with day-0 and NHK samples. To exclude a potential effect of *HIPK4* silencing on other undesired lineage differentiation, we verified that endodermal, mesodermal, and neuroectodermal markers' expression was minimal under *HIPK4* silencing at day 10 compared with the control (Figure S3C and Table S5). A hierarchical clustering of the samples then gave two main points of information (Figure S4A): (1) *HIPK4* knockdown at day 5 induced no major effect as HIPK4 was still not expressed at this time point (Figure 3C); and (2) *HIPK4* knockdown at day 10 is clearly distinct from knockdown at day 5. Moreover, we were able to retrieve in a hierarchical gene clustering pluripotency markers, expression of which was high at day 0 and was then reduced during the differentiation process. Conversely, epithelial and epidermal markers were upregulated during the process and to a higher extent under *HIPK4* knockdown than in the control at day 10 (Figure 3D). As explained above, these markers were not regulated at day 5. Furthermore, principal component analysis showed that differentiated samples were very different from day 0 and NHK samples (Figure S4B).

Finally, we performed a pathway analysis of the top regulated genes under *HIPK4* knockdown at day 10 (Figure 3E and Table S6). Among the top regulated signaling pathways, we found Wnt/β-catenin, NF-κB, PTEN (phosphatase and tensin homolog), interleukin-6 (IL-6), and transforming growth factor β (TGF-β)/BMP. Interestingly, we were able to confirm by qPCR an upregulation of *IL-6* under *HIPK4* knockdown, as well as an upregulation of one TGF-β receptor (*TGFBR2*), although *TGFB1* and one downstream target *CTGF* were not regulated. These data suggest that IL-6 and TGF-β/BMP signaling may be involved in an HIPK4-dependent mechanism (Figure 3F).

Together, loss of *HIPK4* during the early steps of epithelial differentiation leads to a significant enrichment in $K18^+$ cell number and to an increase in epithelial and epidermal gene signature, confirming a negative role of HIPK4 in this process. In addition, loss of *HIPK4* also leads to an increase in $K14^+$ cell number after 30 days of differentiation and to an upregulation of keratinocyte markers. Furthermore, we hypothesize that IL-6 and/or TGF-β signaling could be involved in this mechanism, although this needs to be confirmed by additional investigation.

Loss of HIPK4 Alone Is Not Sufficient to Induce Differentiation but Its Kinase Activity Is Necessary to Upregulate K8/K18

We then tested the effect of the absence of RA and BMP4 in DKSFM medium on the differentiation. First, in the presence of the cytokines no effect of the knockdown was observed at day 5, but the expression of *HIPK4* at day 10 was reduced by the knockdown and K18 expression was 2-fold upregulated (Figures 4A and 4B). Interestingly, the cells maintained in DKSFM medium without RA or BMP4 did not undergo differentiation and remained in stem cell colonies (Figure 4C). qPCR analysis showed that *siHIPK4* had no effect on K18 expression not only at day 5 but also at day 10 compared with the control (Figure 4D). These results indicate that although *HIPK4* silencing contributes to K18 regulation during early epithelial differentiation, it is not enough to induce differentiation from hiPSCs by itself.

To confirm the role of HIPK4 in K18 expression, we overexpressed a wild-type form and a kinase dead mutant of HIPK4 in hiPSCs and differentiated the cells for 10 days. In the control condition, K8 and K18 were upregulated as expected, compared with day 0. Interestingly, the overexpression of wild-type HIPK4 led to a decrease in K8 and K18 expression compared with the control condition (Figure 4E). Moreover, the overexpression of the mutant HIPK4 (kinase dead) restored the expression of K8 and K18 to levels almost similar to the control condition. These data confirm that HIPK4 functions as a brake to epithelial differentiation, and this involves its kinase activity.

HIPK4 Silencing Promotes the Differentiation of Epithelial Precursors in Organotypic Cultures

Lastly, we investigated the effect of epithelial precursors in organotypic cultures (OTCs) by evaluating the epithelial growth and morphogenesis. We expected an optimal induction of epithelial differentiation due to the inductive potential of the dermal equivalent and the air exposure in this 3-dimensional *in vitro* model (Stark et al., 2004). For establishment of OTCs, hiPSCs were transfected with *HIPK4* siRNA, cultured for 4 or 10 days under differentiation conditions, and seeded on scaffold-based dermal equivalents. While a 10-day predifferentiation hampered successful growth in OTCs (data not shown), predifferentiation of 4 days allowed for a sufficient number of cells to attach and grow in OTCs for 3 more weeks (Figure 5A). OTCs were analyzed for epithelial markers by immunohistochemistry. In the *siControl* condition, cells demonstrate organoid structures of mixed populations of mesenchymal cells and K8/K18-positive epithelial cells. In contrast, cells transduced with *HIPK4* siRNA showed an improved differentiation in early epithelium (Figures 5B and S5). The laminin staining indicates the basement membrane zone, on top of which epidermal cells should stratify. In the *siControl* condition, very few cells were stained for K8 or K18 because this differentiation time point was too early to generate epidermal progenitors. In the *siHIPK4* condition, however, we observed a thicker basal layer with basal cell stratification, on top of which

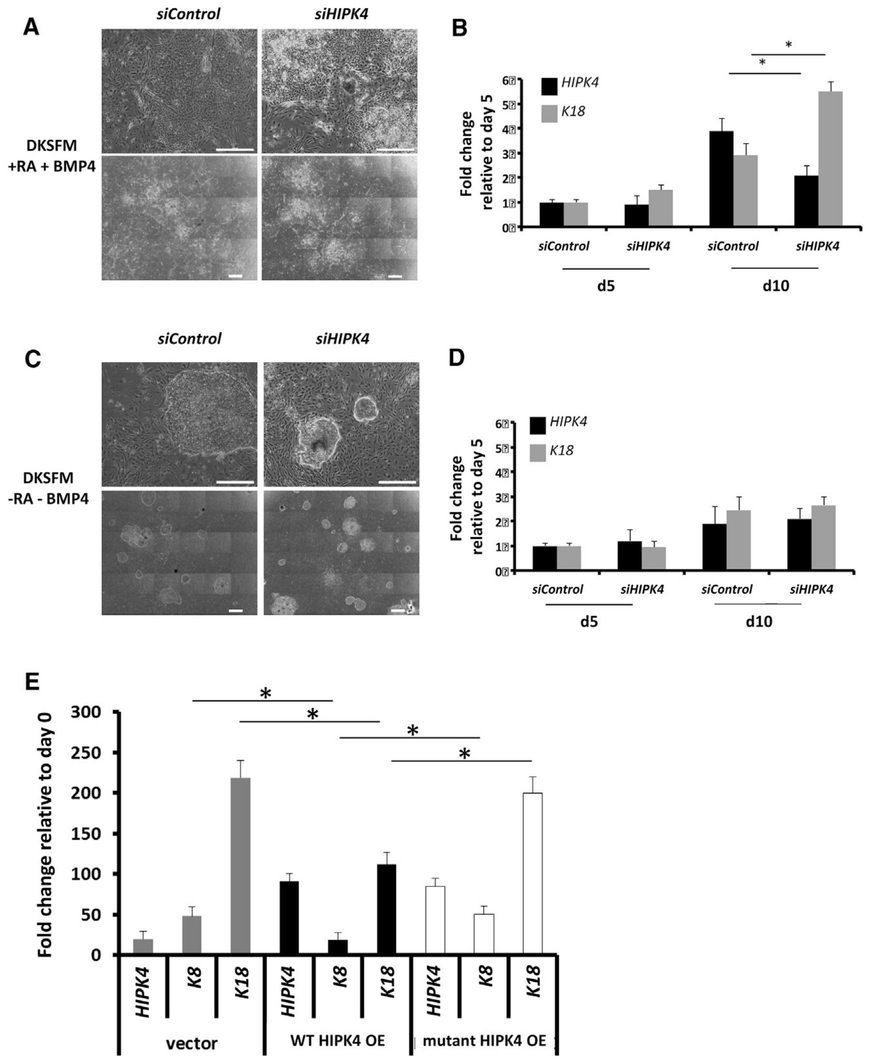

Figure 4. Loss of HIPK4 Alone Is Not Sufficient to Induce Differentiation but Its Kinase Activity Is Necessary to Upregulate K8/K18

(A and C) Bright-field images of cells at day 10 of differentiation in DKSFM medium in the presence (A) or absence (C) of retinoic acid (RA) and bone morphogenetic protein 4 (BMP4) in *siControl* and *siHIPK4* conditions at two different magnifications: top pictures at 20× (scale bar, 600 μm); bottom pictures are a combination of 36 images at 4× (scale bar, 1,000 μm).

(B and D) qPCR analysis of *HIPK4* and *K18* in the presence (B) or absence (D) of RA and BMP4 at day 5 and day 10 is presented. Data represent the mean of three independent experiments ± SEM. Statistical analysis was performed using unpaired Student's t test (*$p < 0.05$).

(E) hiPSCs were transduced with an overexpressing vector for wild-type HIPK4 (WT HIPK4 OE) or kinase dead mutant HIPK4 (mutant HIPK4 OE), or with an empty vector, and differentiated for 10 days. RNA expression of *HIPK4*, *K8*, and *K18* is shown. Data represent a mean of three independent experiments ± SEM. Statistical analysis was performed using unpaired Student's t test (*$p < 0.05$).

lay a monolayer of K8- and K18-positive cells. Moreover, the analysis of later epidermal keratins such as K10 and K14 showed almost no staining in the *siControl* condition but strong staining in the basal layer under *HIPK4* knockdown, suggesting that these keratins were already expressed at this time of differentiation and that a proper basal layer was formed (Figure 5C). Interestingly, the quantification of positive cells for early keratins (K8, K18, K19) and late keratins (K14, K10) showed an increase of all keratin expression whenever *HIPK4* was silenced compared with the control condition (Figure 5C).

DISCUSSION

In this work, we have identified HIPK4 as a key inhibitor of human skin epithelial differentiation by performing a high-throughput RNAi screening analysis during this process and by monitoring the number of K18$^+$ cells. K18 is a classical marker of simple epithelia during early embryonic development, which includes ectodermal epithelium (Baribault et al., 1993). Nevertheless, the results presented here showed an upregulation of ectodermal/skin epithelial markers and no expression of endodermal, mesodermal, or neuroectodermal markers at day 10 of differentiation. These data strongly suggest that K18$^+$ cells monitored in the screen were mainly skin epithelial cells. Interestingly, several members of the MAPK signaling pathway were found among the screen's candidates. This signaling pathway plays a central role in keratinocyte differentiation, since *Map2k1*/*Map2k2* (*Mek1*/*Mek2*) double-knockout mice die 24 hr postnatally, due to dehydration and loss of barrier function of the skin (Scholl et al., 2007). Also, MAPKAPK2 and 3 (mitogen-activated protein kinase-activated protein kinase 2 and 3) are kinases which are regulated by the FGFR signaling pathway (Tan et al., 1996).

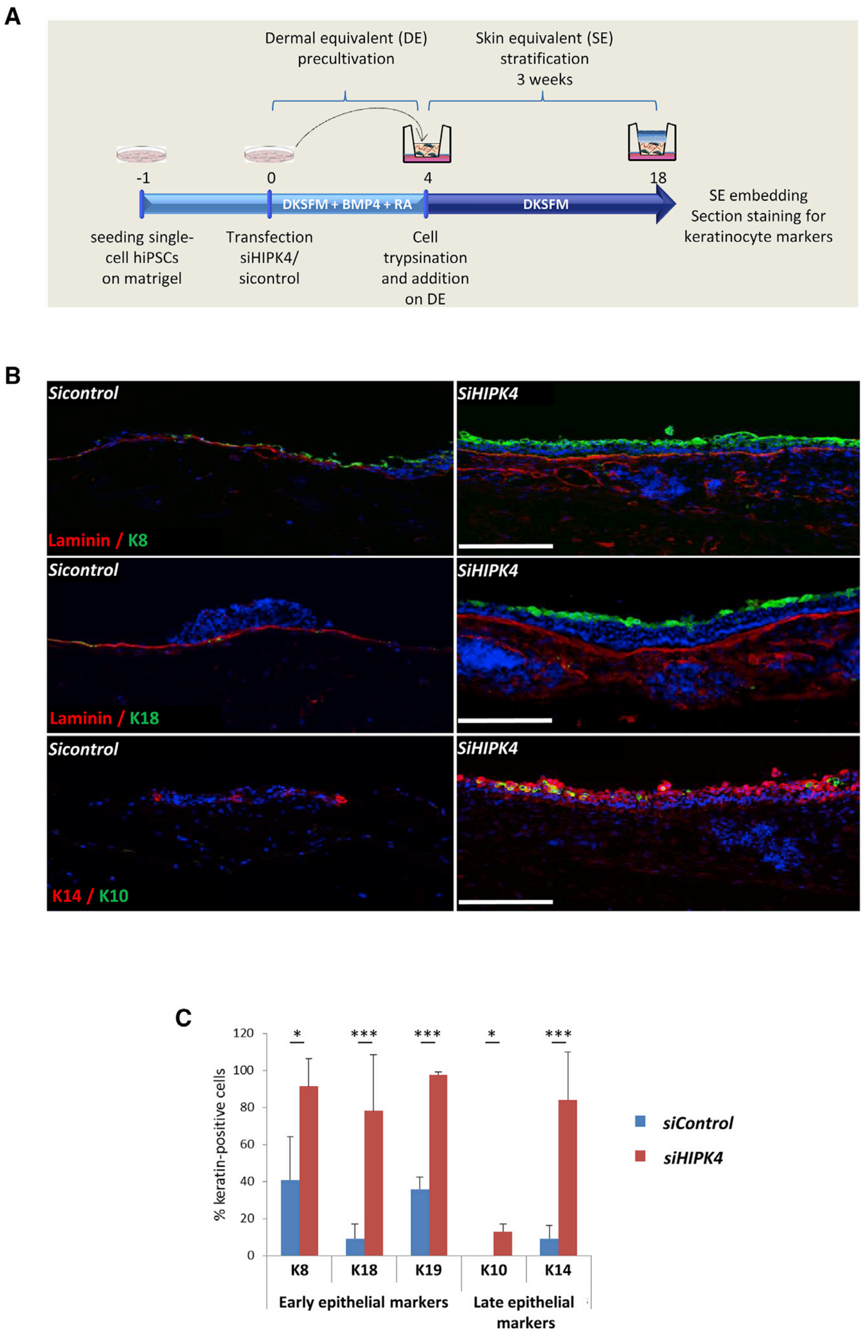

Figure 5. HIPK4 Silencing Promotes the Differentiation of Epithelial Precursors in Organotypic Cultures

(A) Schematic overview of the organotypic culture (OTC) protocol. hiPSC D1 were differentiated for 4 days and included in a 3D co-culture with human dermal fibroblasts for 3 weeks.

(B) Immunostaining against Laminin β1 and K8, K18, K10, and K14 in paraffin sections of 3-week-old OTCs from either HIPK4 siRNA (*siHIPK4*) or a non-targeting siRNA (*siControl*) transfected cells. Scale bar, 100 μm.

(C) Graph showing quantification of keratin-positive cells as a percentage of the total epithelial cells. Antibodies against K14, K10, K19, K8, and K18 were used. Data represent the mean of three independent experiments ± SEM. Statistical analysis was performed using unpaired Student's t test (*$p < 0.05$, ***$p < 0.001$).

HIPK4 regulation has not been extensively studied to date. It was described to phosphorylate p53 at Ser9 and to be structurally related to HIPK1–3 for its catalytic domain (Arai et al., 2007). Although *Hipk1/2* double-knockout mice die in utero, suggesting overlapping functions for these two family members (Isono et al., 2006), no *Hipk4* knockout mouse has been deeply investigated. Based on the transcriptome analysis of the knockdown cells, we suggest that HIPK4 could influence several signaling pathways such as Wnt/β-catenin, NF-κB, PTEN, IL-6, and TGF-β/BMP. Wnt/β-catenin signaling is an important regulator during skin development and, in particular, catenins play a key role in epidermal development (Augustin, 2015; Perez-Moreno and Fuchs, 2006). Interestingly, NF-κB signaling, which was predicted to be inactivated in our analysis, can induce epithelial cell growth arrest (Seitz et al., 1998, 2000). PTEN signaling is involved in epithelial migration, proliferation, and morphogenesis (Castilho et al., 2013;

Georgescu et al., 2014). Although IL-6 signaling (and its downstream target *STAT3*) was recently shown to be involved in lung epithelial development, its role in skin epithelial development is yet to be clarified (Kameyama et al., 2017). Of note, IL-6 can increase the phosphorylation of K18 in epithelial cells (Wang et al., 2007). Lastly, TGF-β/BMP signaling is known to be important in early development and particularly in epidermal differentiation (D'Souza et al., 2001; Li et al., 2003; McDonnell et al., 2001; Park and Morasso, 2002). More recently, inhibition of TGF-β signaling by a small compound was suggested to improve keratinocyte differentiation from hiPSCs (Shalom-Feuerstein et al., 2013).

In conclusion, after validation of a kinome RNAi screen, we demonstrated that loss of *HIPK4* expression improves early skin epithelial differentiation accompanied by an increase in K8 and K18 expression, and improves late keratinocyte differentiation with an increase of epidermal identity (K5/K14/p63). Additionally we found that *HIPK4* knockdown favors the epithelial differentiation in an organotypic culture, with an enhanced basal layer formation and an increased expression of several keratins, including late epidermal keratins such as K14 and K10. Therefore we propose that HIPK4 acts as a control mechanism of epithelial/epidermal differentiation by preventing excessive $K18^+$ precursor differentiation. This mechanism involving HIPK4 kinase activity and phosphorylation of its downstream targets should be examined more closely. This mechanism may also involve the activation of IL-6 and/or TGF-β signaling pathways. Taken together, these data present microscopy RNAi screens as a powerful tool to reveal novel regulators of human skin epithelial differentiation, and therefore will help to improve the current differentiation protocols from PSCs.

EXPERIMENTAL PROCEDURES

Generation of hiPSCs

Human iPSC D1 and HD1 lines were generated from fibroblasts of healthy donors (University Medical Center Mannheim, Germany; ethics committee approval no. 2009-350N-MA) as previously described (Larribere et al., 2015). In brief, human fibroblasts were infected with lentiviral particles carrying an inducible polycistronic cassette containing the reprogramming factors *OCT4*, *SOX2*, *KLF4*, and *c-MYC*. Generated iPSCs were maintained in culture on Matrigel in mTeSR medium (Invitrogen Life Technologies, Darmstadt, Germany).

Differentiation of hiPSCs into Skin Epithelial Cells

For skin epithelial differentiation, the protocol was adapted from Itoh et al. (2011). Cells were trypsinized and seeded at single-cell level in DKSFM containing 1 μM all-*trans* RA, 10 ng/mL BMP4, and 50 μg/mL Normocin. The next day, medium was changed to fresh DKSFM containing RA and BMP4. On day 4, cells were washed twice with DMEM/F12 and fresh DKSFM supplemented with 50 μg/mL Normocin. The differentiation was stopped at different time points for RNA or protein isolation.

High-Throughput RNAi Screen

siRNAs (siGenome, Dharmacon) were prealiquoted in Matrigel-precoated 384-well plates to a final concentration of 250 nM using an automated workstation (Biomek FX, Beckman Coulter). hiPSCs were reverse transfected using Lipofectamine RNAiMAX Transfection Reagent (Life Technologies). After trypsinization, cells were seeded either with a cytokine-containing DKSFM (for differentiation) or with mTeSR (for pluripotency maintenance). The plates were shortly spun down and kept at 37°C and 5% CO_2. On culture day 5 an additional 50 μL of DKSFM without additional cytokines was added. The cells were cultured until day 10. Fixation (4% paraformaldehyde), blocking (3% BSA and 0.05% Triton X-100), and fluorescence staining were performed using an automated workstation. The K18 primary antibody dilution was 1:25. The plates were sealed and incubated over night at 4°C. Next day the cells were washed three times with PBS and incubated for 1 hr with the secondary antibody (anti-mouse Atto 594) together with Hoechst. After washing, the plates were sealed and stored at 4°C until imaging. The high-throughput screen was performed using a human RNAi library (siGenome, Dharmacon) consisting of a pool of four siRNAs per gene, targeting kinases, and kinase-regulatory proteins.

Image Analysis of Screen Data

Imaging was performed using an automated BD Pathway 855 Bioimaging System (Becton Dickinson) with a 20× objective (numerical aperture 0.75) and a Hamamatsu digital camera (Orca-ER). Per well, 25 fields were imaged for both filters, Hoechst (nuclei staining) and Atto 594 (K18 staining). Images were analyzed using the Cellprofiler image analysis software Version 2.0. Object selection was based on adaptive intensity and fixed-size thresholds for each individual object. The segmentation of objects was optimized in order to reach the best resolution even in dense cell clusters. For data analysis, parent objects were segmented in channel 1 (Hoechst), and in channel 2 (Atto 594) the mean intensity of child objects was measured. Based on the mean intensity of every nucleus, nuclei were categorized into a $K18^+$ and $K18^-$ cell, according to the manually set mean intensity threshold. The values of $K18^+$ cells were used for further analysis. To prevent strong viability effects, we excluded wells with less than 1,000 cells from the analysis. For data analysis, cellHTS2 software was used. The fraction of $K18^+$ cells per well was normalized per assay plate and the *Z* scores were calculated using the R package cellHTS2. The correlation between the data of replicates was estimated using the Spearman rank correlation coefficient. Inhibitor hit: *Z* score < positive control; promoter hit: *Z* score > 2.

Gene Expression and Ingenuity Analyses

Illumina gene expression raw data from biological triplicates of NHK, D1 hiPSC (day 0), day-5, day-10, day-30 differentiated cells, and their counterpart with silenced *HIPK4* were normalized in CHIPSTER software. Unsupervised hierarchical clustering was performed after filtering genes with a variance test ($p < 0.05$).

Differential gene expression between day-10 *siHIPK4* sample and day-10 *siControl* sample was determined using an empirical Bayes statistical test. Gene signatures for ectodermal development or epithelial and epidermal differentiation were downloaded from the GO database (GO:0030856, GO:0072148). Gene signatures for pluripotency, or endodermal, mesodermal, and neuroectodermal development were downloaded from the GO database or from published transcriptome profiling (GO:0019827) (Tadeu et al., 2015).

Regulated genes (n = 391) in day-10 *siHIPK4* sample were then uploaded to IPA (Ingenuity Pathway Analysis) software to evaluate the most regulated signaling pathways. All raw data have been uploaded on the public database under accession GEO: GSE102067.

siRNA Transfection

The cells were transfected 1 day after seeding and differentiated for 10 days. Transfection reagent (Lipofectamine RNAiMAX) was added to DMEM/F12 and incubated for 10 min. After incubation time, either the set of four siRNAs against *HIPK4* or the non-targeting *siControl* were added and incubated for 20 min at room temperature. The transfection mix was then added to the cells with a final siRNA concentration of 25 nM. After 2 days, medium was aspirated, the cells were washed twice with PBS, and cytokine-containing DKSFM was added. At day 4 of differentiation the medium was aspirated, and the cells washed again and cultured in DKSFM without cytokines. The medium was changed every 2 days. siRNA sequences were: *HIPK4* J-004808-09 AGU AUA UGC UCA AGU CGU U; *HIPK4* J-004808-10 AGA CGA AGG UGC GCC CAU U; *HIPK4* J-004808-11 AGA AGG AGG CUG CGG GUA U; *HIPK4* J-004808-12 GCA ACA ACG AGU ACG ACC A; Non-targeting Pool #2 (1) UAA GGC UAU GAA GAG AUA C; Non-targeting Pool #2 (2) AUG UAU UGG CCU GUA UUA G; Non-targeting Pool #2 (3) AUG AAC GUG AAU UGC UCA A; Non-targeting Pool #2 (4) UGG UUU ACA UGU CGA CUA A.

Overexpression

Human iPSCs were transduced with a lentiviral expression vector (pTriEx-1) coding for wild-type *HIPK4* (WT HIPK4 OE), kinase dead mutant *HIPK4* (mutant HPIK4), or with the empty vector (vector) as a control, and differentiated for 10 days.

Immunofluorescence

Cells grown on coverslips were washed twice with PBS, fixed with 4% paraformaldehyde in PBS for 30 min, and treated with blocking buffer (3% BSA, 0.1% Triton X-100 in 1× PBS) for 45 min. Antibody dilution (500 μL) was added per coverslip and incubated overnight at 4°C. Primary antibodies included keratin-14 (Covance), keratin-18 (Progen), and p63 (Abcam). Quantification of positive cells was calculated after counting a minimum of 300 cells per conditions.

Western Blot and qPCR

Whole-cell extracts representative of three independent experiments were prepared from hiPSCs, NHK isolated from biopsies of healthy donors (University Medical Center Manheim, Germany), or differentiated cells at different time points. Primary antibodies used were K18 (Progen, #11,416), HIPK4 (Acris, #AP52050PU-N), and α-actinin (Santa Cruz Biotechnology, #sc-17829). RNA from hiPSCs, NHK, or from day-5, day-10, and day-30 differentiated cells was extracted using an RNeasy kit (QIAGEN). Real-time qPCR was performed using the SYBR Green Supermix (Applied Biosystems) on a 7500 Real-Time PCR system (Applied Biosystems). Primers used for qPCR were as follows: *18S*, 5′-GAG GAT GAG GTG GAA CGT GT-3′ (forward [fwd]) and 5′-TCT TCA GTC GCT CCA GGT CT-3′ (reverse [rev]); *GAPDH*, 5′-GAA GGT GAA GGT CGG AGT C-3′ (fwd) and 5′-GAA GAT GGT GAT GGG ATT TC-3′ (rev); *K14*, 5′-AGG AGA TCG CCA CCT ACC GCC-3′ (fwd) and 5′-AGG AGG TCA CAT CTC TGG ATG ACT G-3′ (rev); *K18*, 5′-GAG TAT GAG GCC CTG CTG AAC ATC A-3′ (fwd) and 5′-GCG GGT GGT GGT CTT TTG GAT-3′ (rev); *K5*, 5′-ATC TCT GAG ATG AAC CGG ATG ATC-3′ (fwd) and 5′-CAG ATT GGC GCA CTG TTT CTT-3′ (rev); *K8*, 5′-GAT CGC CAC CTA CAG GAA GCT-3′ (fwd) and 5′-ACT CAT GTT CTG CAT CCC AGA CT-3′ (rev); *p63*, 5′-TTC TTA GCG AGG TTG GGC TG-3′ (fwd) and 5′-GAT CGC ATG TCG AAA TTG CTC-3′ (rev), Involucrin, 5′-CTC CAT GTG TCA TGG GAT ATG-3′ (fwd) and 5′-TCA ACC TGA AAG ACA GAA GAG-3′ (rev). Expression values were normalized to housekeeping genes *18S* or *GAPDH*.

Organotypic Culture and Immunohistochemistry

The dermal equivalents were made in membrane insert-containing 6-well plates and comprised a circular piece of non-woven scaffold (22 mm), 750 μL of thrombin (10 U/mL) mixed with human dermal fibroblasts in fetal bovine serum (FBS), and 750 μL of fibrinogen (8 mg/mL). Thrombin and fibroblasts were soaked into the dry scaffold before the fibrinogen solution was added carefully and gently mixed. After combining the components, a soft fibrin clot formed in about 10 min at 37°C, which was then immersed in DMEM with 10% FBS containing 50 μg of L-ascorbic acid (sigma) and 1 ng/mL TGFB1 (R&D Systems). Precultivation was performed for 2–5 days with medium change every other day. The day before the epithelial cells were seeded, the dermal equivalents were shifted to rFAD medium (three-fourths DMEM and one-fourth Ham's F12 supplemented with 10% FBS, 10^{-10} M cholera toxin, 0.4 mg of hydrocortisone, and 50 mg/mL L-ascorbic acid) mixed with DKSFM (1:1) to equilibrate them for the co-culture phase. In the organotypic co-cultures, the DKSFM was changed every other day during a period of 3 weeks. Specimens of OTCs were then embedded in Tissue Tek (Miles, Elkhart, IN, USA) and frozen in liquid nitrogen vapor. Cryostat sections (6 μm) were mounted on 3-aminopropyl-triethoxysilane-coated slides and air dried. The sections were fixed in 80% methanol at 4°C for 5 min followed by absolute acetone at −20°C for 2 min and preblocked with 3% BSA in PBS. The incubation with the primary antibody was carried out in a moist chamber either overnight at 4°C or at 37°C for 1 hr followed by 30 min at room temperature. Laminin antibody was gift from Prof. J.M. Foidart, University of Liege, Belgium. Reference numbers of other antibodies: Keratin 14 (BioLegend Inc., #905301), Keratin 10 (Progen, DE-K10, #11414), Keratin 8 (Progen, clone Ks 8.7, #61038), Keratin 18 (Progen, Ks 18.04, #61028), Keratin 19 (Progen, #GP-CK19). Secondary antibodies were applied together with 2 μg/mL DAPI for nuclear staining for 30 min at 37°C followed by 30 min at room temperature in a moist chamber. After washing thoroughly, the sections were mounted in Fluorescent Mounting Medium (Dako) and stored in the dark at 4°C.

The specimens were examined with a Leica DMRBE/RD photomicroscope equipped with epifluorescence illumination, and micrographs were recorded with a CCD camera (F-View 12) applying Analysis Pro 6.0 software (Olympus Soft Imaging Solutions, Munster, Germany).

SUPPLEMENTAL INFORMATION

Supplemental Information includes five figures and six tables and can be found with this article online at http://dx.doi.org/10.1016/j.stemcr.2017.08.023.

AUTHOR CONTRIBUTIONS

Conception and Design, L.L., M.G., P.B., and J.U. Collection and/or Assembly of Data, L.L., M.G., D.N., K.A., H.C.V., H.-J.S., and J.U. Data Analysis and Interpretation, L.L., M.G., D.N., K.A., H.C.V., H.-J.S., P.B., M.B., and J.U. Provision of Study Material or Patients, J.U. Manuscript writing, L.L. and J.U. Final approval of manuscript, L.L., M.G., D.N., K.A., H.C.V., H.-J.S., P.B., M.B., and J.U. Financial support, P.B. and J.U. Administrative support, J.U.

ACKNOWLEDGMENTS

We would like to thank Jenny Dworacek, Daniel Roth, Tatjana Wüst, and Iris Martin for their excellent technical assistance. We also would like to thank Pierre-Olivier Frappart for generously providing mouse embryo sections. This work was supported by grants from the German Research Council (RTG2099 and SFB873 to J.U.) and the Baden-Württemberg Foundation (P-LS-ASII/28 to J.U. and P-BWS-ASII/35 to P.B.).

Received: January 31, 2017
Revised: August 28, 2017
Accepted: August 29, 2017
Published: September 28, 2017

REFERENCES

Aberdam, D. (2004). Derivation of keratinocyte progenitor cells and skin formation from embryonic stem cells. Int. J. Dev. Biol. *48*, 203–206.

Arai, S., Matsushita, A., Du, K., Yagi, K., Okazaki, Y., and Kurokawa, R. (2007). Novel homeodomain-interacting protein kinase family member, HIPK4, phosphorylates human p53 at serine 9. FEBS Lett. *581*, 5649–5657.

Augustin, I. (2015). Wnt signaling in skin homeostasis and pathology. J. Dtsch. Dermatol. Ges. *13*, 302–306.

Baribault, H., Price, J., Miyai, K., and Oshima, R.G. (1993). Id-gestational lethality in mice lacking. Genes Dev. *7*, 1191–1202.

Botchkarev, V.a, Botchkareva, N.V., Roth, W., Nakamura, M., Chen, L.H., Herzog, W., Lindner, G., McMahon, J.a, Peters, C., Lauster, R., et al. (1999). Noggin is a mesenchymally derived stimulator of hair-follicle induction. Nat. Cell Biol. *1*, 158–164.

Carvajal-Vergara, X., Sevilla, A., D'Souza, S.L., Ang, Y.-S., Schaniel, C., Lee, D.-F., Yang, L., Kaplan, A.D., Adler, E.D., Rozov, R., et al. (2010). Patient-specific induced pluripotent stem-cell-derived models of LEOPARD syndrome. Nature *465*, 808–812.

Castilho, R., Squarize, C., and Gutkind, J. (2013). Exploiting PI3K/mTOR signaling to accelerate epithelial wound healing. Oral Dis. *19*, 551–558.

Coraux, C., Hilmi, C., Rouleau, M., Spadafora, A., Hinnrasky, J., Ortonne, J.P., Dani, C., and Aberdam, D. (2003). Reconstituted skin from murine embryonic stem cells. Curr. Biol. *13*, 1317–1323.

D'Souza, S.J., Pajak, A., Balazsi, K., and Dagnino, L. (2001). Ca2+ and BMP-6 signaling regulate E2F during epidermal keratinocyte differentiation. J. Biol. Chem. *276*, 23531–23538.

Erfle, H., Neumann, B., Liebel, U., Rogers, P., Held, M., Walter, T., Ellenberg, J., and Pepperkok, R. (2007). Reverse transfection on cell arrays for high content screening microscopy. Nat. Protoc. *2*, 392–399.

Galach, M., and Utikal, J. (2011). From skin to the treatment of diseases–the possibilities of iPS cell research in dermatology. Exp. Dermatol. *20*, 523–528.

Georgescu, M.-M., Cote, G., Agarwal, N.K., and White, C.L. (2014). NHERF1/EBP50 controls morphogenesis of 3D Colonic glands by stabilizing PTEN and ezrin-radixin-moesin proteins at the apical membrane. Neoplasia *16*, 365–374.e2.

Green, H., Easley, K., and Iuchi, S. (2003). Marker succession during the development of keratinocytes from cultured human embryonic stem cells. Proc. Natl. Acad. Sci. USA *100*, 15625–15630.

Guenou, H., Nissan, X., Larcher, F., Feteira, J., Lemaitre, G., Saidani, M., Del Rio, M., Barrault, C.C., Bernard, F.X., Peschanski, M., et al. (2009). Human embryonic stem-cell derivatives for full reconstruction of the pluristratified epidermis: a preclinical study. Lancet *374*, 1745–1753.

He, Q., Shi, J., Sun, H., An, J., Huang, Y., and Sheikh, M.S. (2010). Characterization of human homeodomain-interacting protein kinase 4 (HIPK4) as a unique member of the HIPK family. Mol. Cell Pharmacol. *2*, 61–68.

Hu, Y., Baud, V., Oga, T., Kim, K.I., Yoshida, K., and Karin, M. (2001). IKKa controls formation of the epidermis independently of NF-kB. Nature *410*, 710–714.

Isono, K., Nemoto, K., Li, Y., Takada, Y., Suzuki, R., Katsuki, M., Nakagawara, A., and Koseki, H. (2006). Overlapping roles for homeodomain-interacting protein kinases hipk1 and hipk2 in the mediation of cell growth in response to morphogenetic and genotoxic signals. Mol. Cell Biol. *26*, 2758–2771.

Itoh, M., Kiuru, M., Cairo, M.S., and Christiano, A.M. (2011). Generation of keratinocytes from normal and recessive dystrophic epidermolysis bullosa-induced pluripotent stem cells. Proc. Natl. Acad. Sci. USA *108*, 8797–8802.

Iuchi, S., Dabelsteen, S., Easley, K., Rheinwald, J.G., and Green, H. (2006). Immortalized keratinocyte lines derived from human embryonic stem cells. Proc. Natl. Acad. Sci. USA *103*, 1792–1797.

Kameyama, H., Kudoh, S., Hatakeyama, J., Matuo, A., and Ito, T. (2017). Significance of Stat3 signaling in epithelial cell differentiation of fetal mouse lungs. Acta Histochem. Cytochem *50*, 1–9.

Kondo, T., Asai, M., Tsukita, K., Kutoku, Y., Ohsawa, Y., Sunada, Y., Imamura, K., Egawa, N., Yahata, N., Okita, K., et al. (2013). Modeling Alzheimer's disease with iPSCs reveals stress phenotypes associated with intracellular Aβ and differential drug responsiveness. Cell Stem Cell *12*, 487–496.

Larribere, L., Wu, H., Novak, D., Galach, M., Bernhardt, M., Orouji, E., Weina, K., Knappe, N., Sachpekidis, C., Umansky, L., et al. (2015). NF1 loss induces senescence during human melanocyte differentiation in an iPSC-based model. Pigment Cell Melanoma Res. *28*, 407–416.

Li, A.G., Koster, M.I., and Wang, X.J. (2003). Roles of TGFbeta signaling in epidermal/appendage development. Cytokine Growth Factor. Rev. *14*, 99–111.

McDonnell, M.A., Law, B.K., Serra, R., and Moses, H.L. (2001). Antagonistic effects of TGFbeta1 and BMP-6 on skin keratinocyte differentiation. Exp. Cell Res. *263*, 265–273.

Metallo, C.M., Ji, L., de Pablo, J.J., and Palecek, S.P. (2008). Retinoic acid and bone morphogenetic protein signaling synergize to efficiently direct epithelial differentiation of human embryonic stem cells. Stem Cells *26*, 372–380.

Nissan, X., Larribere, L., Saidani, M., Hurbain, I., Delevoye, C., Feteira, J., Lemaitre, G., Peschanski, M., and Baldeschi, C. (2011). Functional melanocytes derived from human pluripotent stem cells engraft into pluristratified epidermis. Proc. Natl. Acad. Sci. USA *108*, 14861–14866.

Panchision, D., and Pickel, J. (2001). Sequential actions of BMP receptors control neural precursor cell production and fate. Genes Dev. *6*, 2094–2110.

Park, G.T., and Morasso, M.I. (2002). Bone morphogenetic protein-2 (BMP-2) transactivates Dlx3 through Smad1 and Smad4: alternative mode for Dlx3 induction in mouse keratinocytes. Nucleic Acids Res. *30*, 515–522.

Perez-Moreno, M., and Fuchs, E. (2006). Catenins: keeping cells from getting their signals crossed. Dev. Cell *11*, 601–612.

Raya, A., Rodríguez-Pizà, I., Guenechea, G., Vassena, R., Navarro, S., Barrero, M.J., Consiglio, A., Castellà, M., Río, P., Sleep, E., et al. (2009). Disease-corrected haematopoietic progenitors from Fanconi anaemia induced pluripotent stem cells. Nature *460*, 53–59.

Scholl, F.A., Dumesic, P.A., Barragan, D.I., Harada, K., Bissonauth, V., Charron, J., and Khavari, P.A. (2007). Mek1/2 MAPK kinases are essential for mammalian development, homeostasis, and raf-induced hyperplasia. Dev. Cell *12*, 615–629.

Seitz, C.S., Lin, Q., Deng, H., and Khavari, P.A. (1998). Alterations in NF-kappaB function in transgenic epithelial tissue demonstrate a growth inhibitory role for NF-kappaB. Proc. Natl. Acad. Sci. USA *95*, 2307–2312.

Seitz, C.S., Deng, H., Hinata, K., Lin, Q., and Khavari, P.A. (2000). Nuclear factor kappaB subunits induce epithelial cell growth arrest. Cancer Res. *60*, 4085–4092.

Shalom-Feuerstein, R., Serror, L., Aberdam, E., Müller, F.-J., van Bokhoven, H., Wiman, K.G., Zhou, H., Aberdam, D., and Petit, I. (2013). Impaired epithelial differentiation of induced pluripotent stem cells from ectodermal dysplasia-related patients is rescued by the small compound APR-246/PRIMA-1MET.SUPP. Proc. Natl. Acad. Sci. USA *110*, 2152–2156.

Stark, H.-J., Willhauck, M.J., Mirancea, N., Boehnke, K., Nord, I., Breitkreutz, D., Pavesio, A., Boukamp, P., and Fusenig, N.E. (2004). Authentic fibroblast matrix in dermal equivalents normalises epidermal histogenesis and dermoepidermal junction in organotypic co-culture. Eur. J. Cell Biol. *83*, 631–645.

Tadeu, A.M.B., Lin, S., Hou, L., Chung, L., Zhong, M., Zhao, H., and Horsley, V. (2015). Transcriptional profiling of ectoderm specification to keratinocyte fate in human embryonic stem cells. PLoS One *10*, e0122493.

Tan, Y., Rouse, J., Zhang, A., Cariati, S., Cohen, P., and Comb, M.J. (1996). FGF and stress regulate CREB and ATF-1 via a pathway involving p38 MAP kinase and MAPKAP kinase-2. EMBO J. *15*, 4629–4642.

Tolar, J., Xia, L., Riddle, M.J., Lees, C.J., Eide, C.R., McElmurry, R.T., Titeux, M., Osborn, M.J., Lund, T.C., Hovnanian, A., et al. (2011). Induced pluripotent stem cells from individuals with recessive dystrophic epidermolysis bullosa. J. Invest. Dermatol. *131*, 848–856.

Wang, L., Srinivasan, S., Theiss, A.L., Merlin, D., and Sitaraman, S.V. (2007). Interleukin-6 induces keratin expression in intestinal epithelial cells. J. Biol. Chem. *282*, 8219–8227.

Stem Cell Reports, Volume *9*

Supplemental Information

An RNAi Screen Reveals an Essential Role for HIPK4 in Human Skin Epithelial Differentiation from iPSCs

Lionel Larribère, Marta Galach, Daniel Novak, Karla Arévalo, Hans Christian Volz, Hans-Jürgen Stark, Petra Boukamp, Michael Boutros, and Jochen Utikal

A.

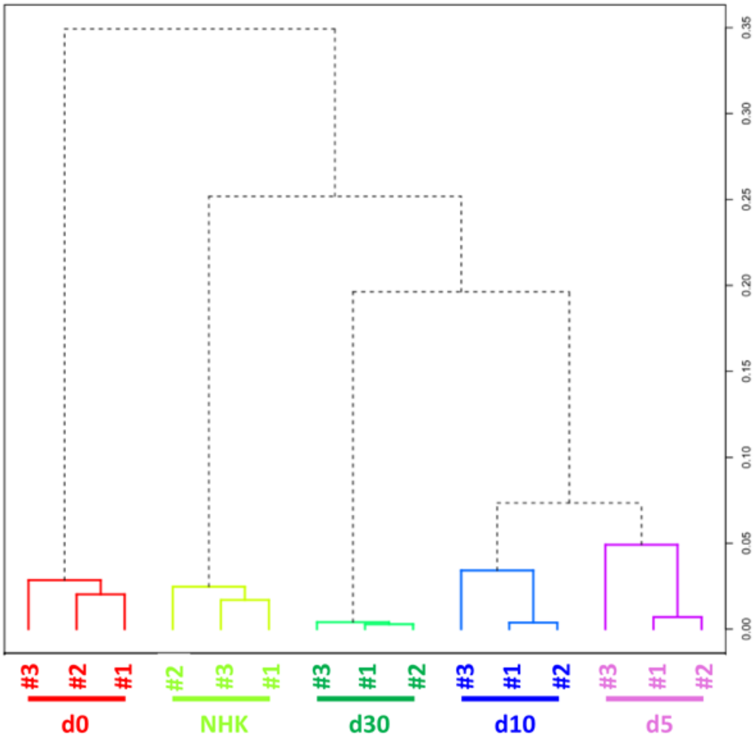

B.

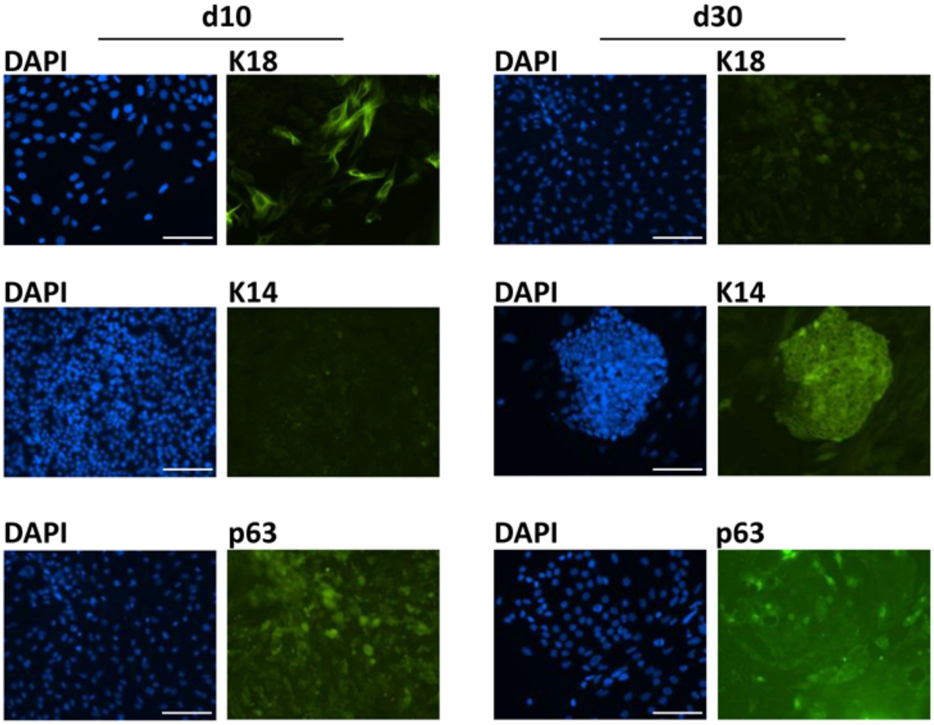

Figure S1.

A. **Related to Figure 1D:** Dendrogram of samples using normalized data with Pearson correlation and average linkage method. The branches of the tree are colored according to the groups: hiPSCs (**d0**), normal human keratinocytes (**NHK**), day 5 (**d5**), day 10 (**d10**) and day 30 (**d30**) differentiated cells. Clustering is done using the function hcluster in which the parameter correlation envokes computation of pearson type of distances.

B. **Related to Figure 1E:** Immunostaining of keratin 18 (K18), keratin 14 (K14) and p63 at day 10 and day 30 of differentiation. Nuclei were stained with DAPI. Scale bar : 100um

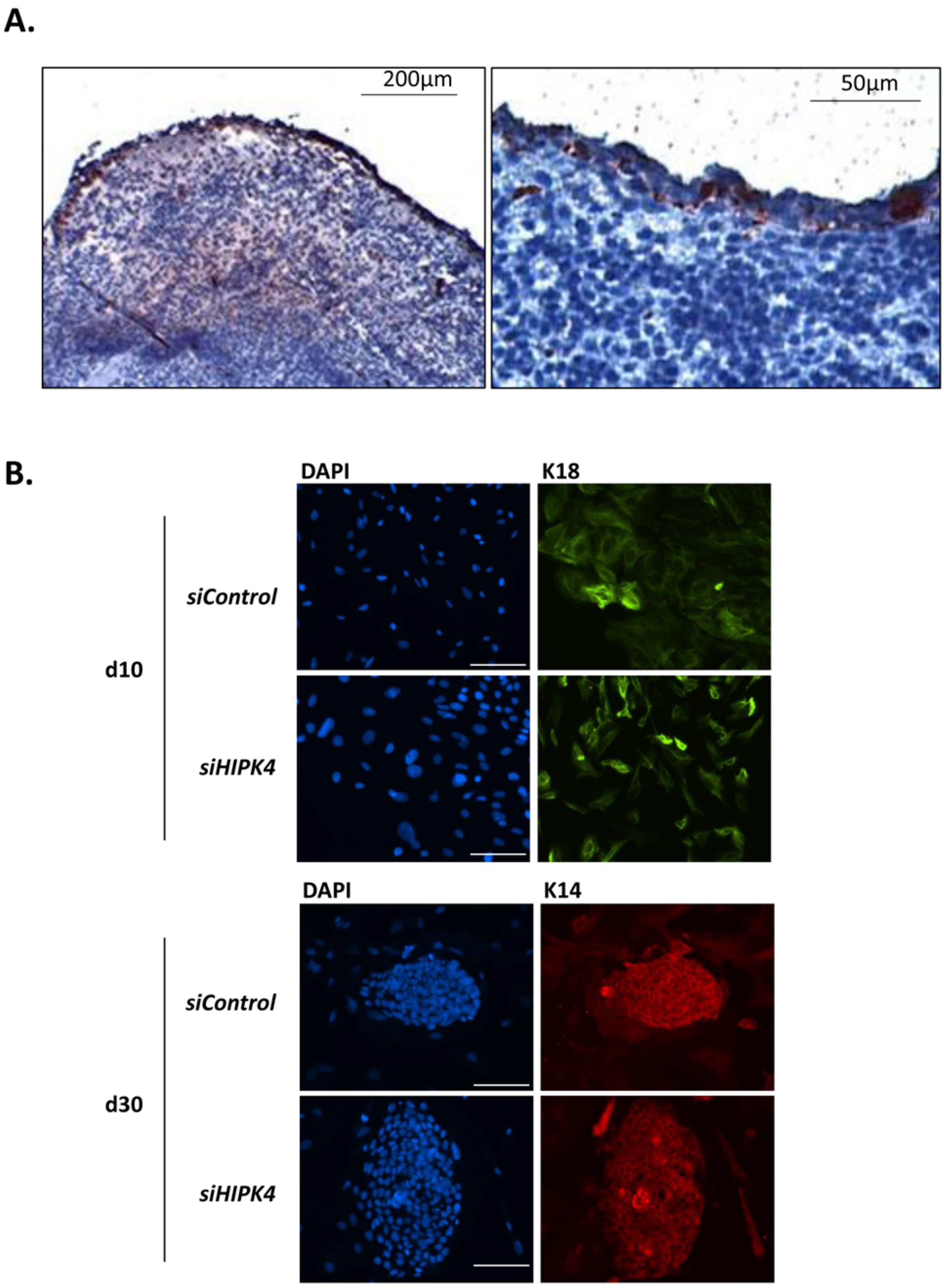

Figure S2.

A. **Related to Figure 2C:** HIPK4 in vivo expression in skin sections of mouse embryos at day E17.5 in the brain region.

B. **Related to Figure 3A:** Upregulation of K18 and K14 under HIPK4 silencing. Immunofluorescence staining against K18 at day 10 and K14 at day 30 of differentiation in control condition (*siControl*) and under HIPK4 knockdown (*siHIPK4*). Scale bar: 100 µm.

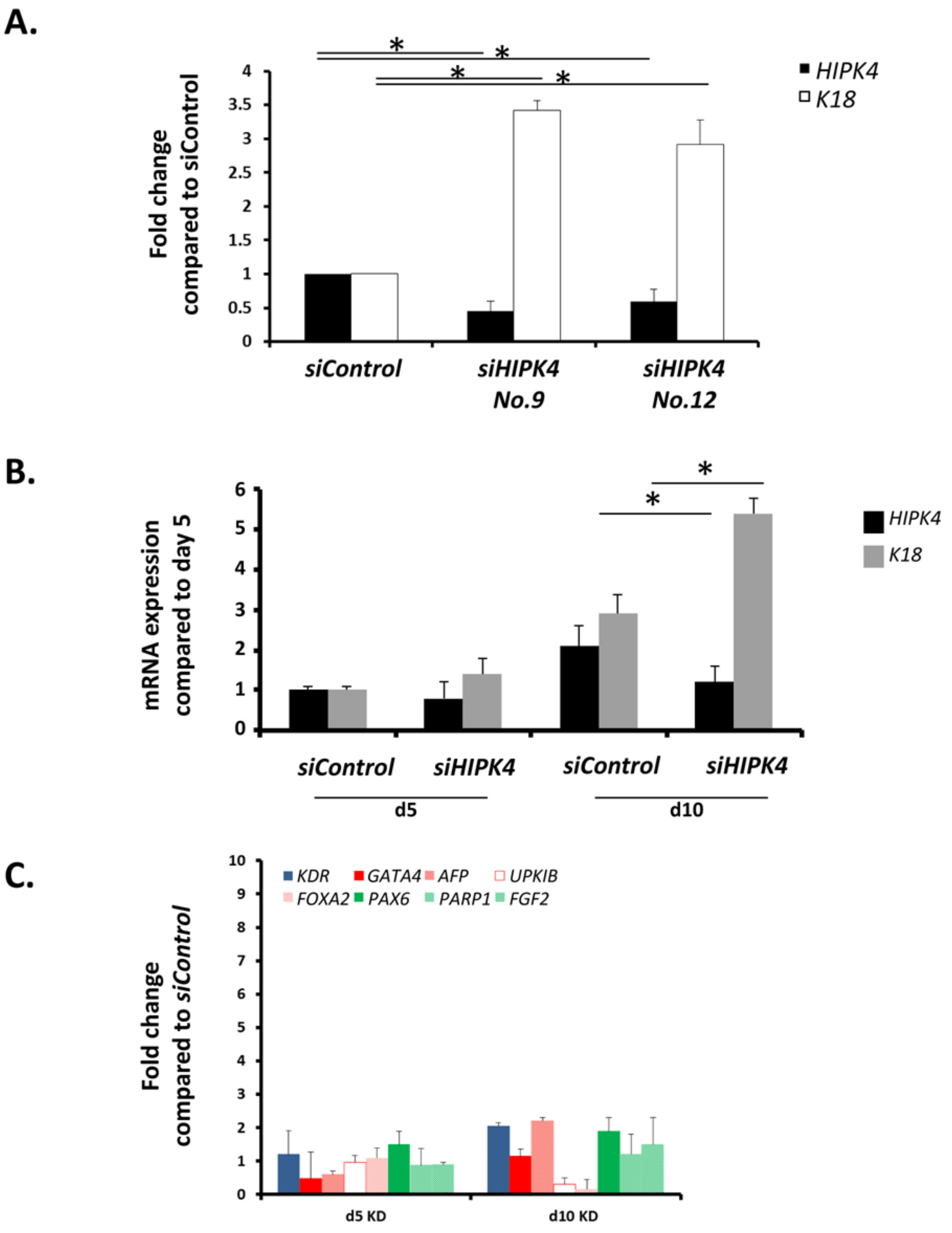

Figure S3.

A. Related to Figure 3C: *HIPK4* silencing with single siRNAs. qPCR analysis of *HIPK4* and Keratin 18 expression under HIPK4 knockdown with single siRNAs (*siNo.9* and *siNo.12*) or in control condition (*siControl*) at day 10 of differentiation. Data represent a mean of three independent experiments ± SEM. Statistical analysis was performed using unpaired Student's t-test (* P<0.05).

B. Related to Figure 3C: qPCR analysis of HIPK4 and K18 expression in a second hiPSC line (HD1) under HIPK4 silencing (*siHIPK4*) or in control condition (*siControl*) at day 5 and day 10 of differentiation. Data represent a mean of three independent experiments ± SEM. Statistical analysis was performed using unpaired Student's t-test (* P<0.1).

C. Related to Figure 3E: qPCR analysis of mesodermal marker (*KDR*), endodermal markers (*GATA4, AFP, UPK1B and FOXA2*) and neuroectodermal markers (*PAX6, PARP1 and FGF2*) at day 5 and day 10 under *HIPK4* knockdown. Data represent a mean of three independent experiments ± SEM.

A.

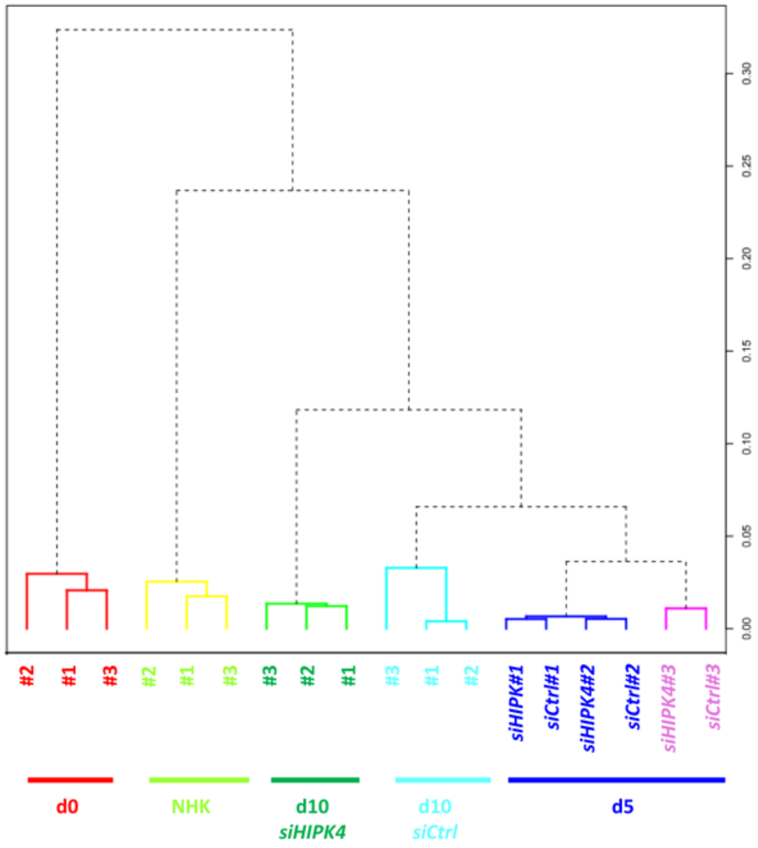

B.

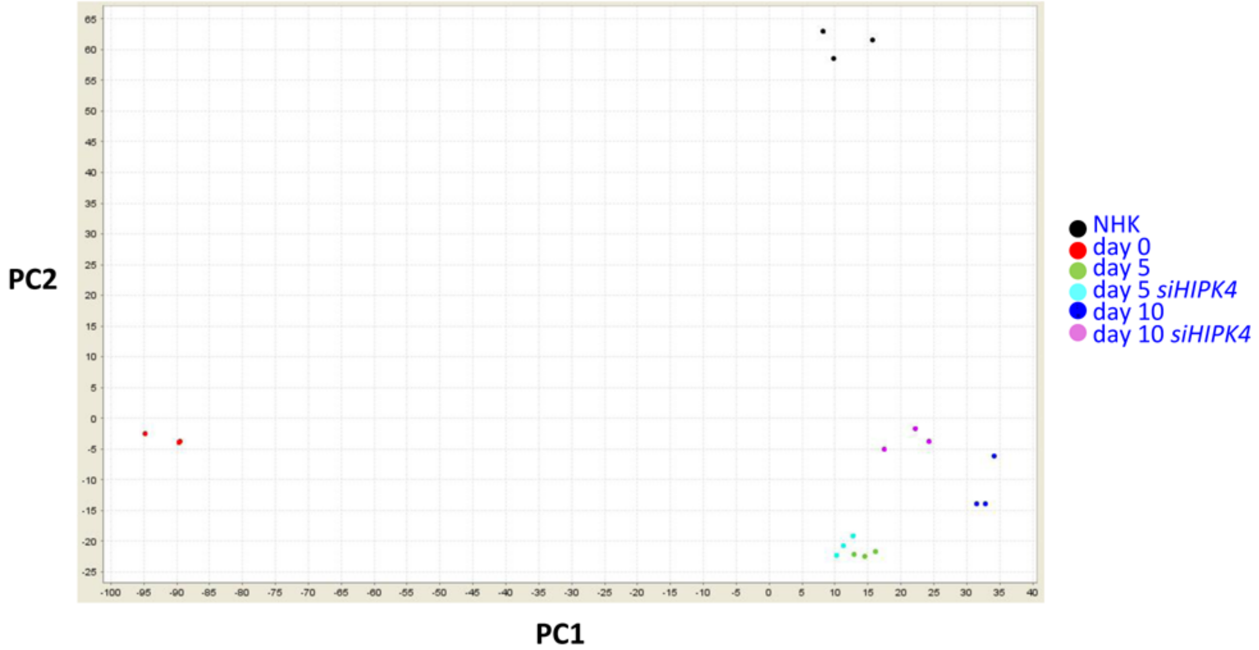

Figure S4. Related to Figure 3E: Transcriptome analysis of *HIPK4* knockdown and control samples at day 5 and day 10 of differentiation.

A. Dendrogram of samples using normalized data with Pearson correlation and average linkage method. The branches of the tree are colored according to the groups: hiPSCs (**d0**), normal human keratinocytes (**NHK**), day 5 (**d5**) and day 10 (**d10**) differentiated cells under control or HIPK4 knockdown conditions. Clustering is done using the function hcluster in which the parameter correlation envokes computation of pearson type of distances.

B. Principal component analysis of gene expression profiles on the same samples as above. Proportion of Variance PC1: 0.44 and PC2: 0.21

A.

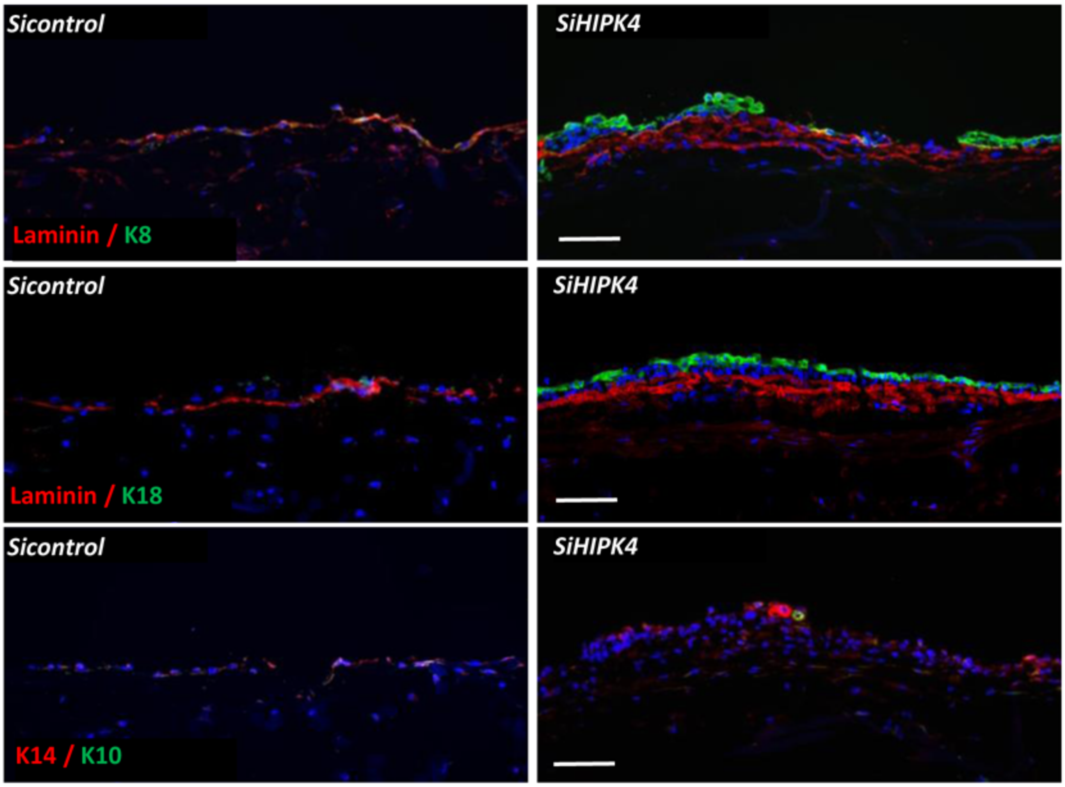

B.

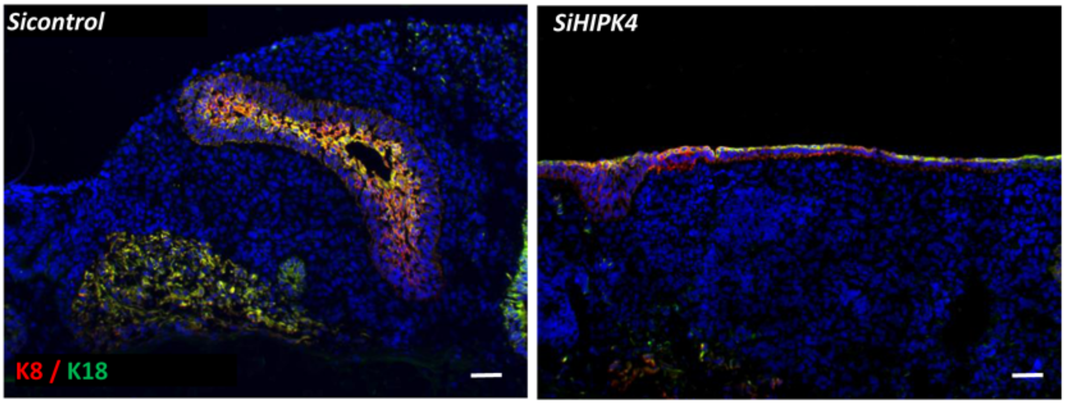

Figure S5. Related to Figure 5: *HIPK4* knockdown promotes epithelial differentiation of coculture with human dermal fibroblasts.

A. Immunostaining against Laminin b1, Keratin 8, 18, 10 and 14 in paraffin sections of 13-day old organotypic cultures (OTCs) from either *HIPK4* siRNA (*siHIPK4*) or a non-targeting siRNA (*siControl*) transfected cells. Scale bar: 100um.

B. Immunostaining against Keratin 8 and 18 in paraffin sections of 21-day old organotypic cultures (OTCs) from either HIPK4 siRNA (*siHIPK4*) or a non-targeting siRNA (*siControl*) transfected cells. OTCs of *HIPK4* siRNA treated cells show an improved directed differentiation. In contrast non-targeting siRNA treated cells demonstrate organoid structures of mixed populations of mesenchymal and epithelial cells. Scale bar: 100µm.

Table S1. Related to Figure 1B: Gene signature of endodermal, mesodermal, neuroectodermal tissue and pluripotency at day 10 of differentiation.

ENDODERM		
symbol	day 5	day 10
AFP	-1.09	-1.77
CD36	-0.04	-0.01
CDX2	0.81	0.02
CLDN6	-0.39	0.41
CTNNBL1	0.26	0.22
CXCR4	-0.01	-0.12
EOMES	-0.70	-0.76
FABP1	-0.06	-0.06
FOXA2	-0.05	-0.08
GATA4	0.04	0.03
GATA6	0.47	0.27
GDF3	-0.98	-1.01
HNF1B	-0.06	-0.11
HNF4A	0.06	0.10
MIXL1	-0.35	-0.31
PECAM1	-0.06	-0.08
SALL4	-1.88	-2.57
SOX1	-0.13	-0.07
SOX17	-0.17	-0.03
UPK1B	0.06	0.03
VEGFA	-0.31	-0.11
VEGFB	0.04	0.10
VEGFC	0.45	0.29

MESODERM		
symbol	day 5	day 10
ACVR1	0.15	-0.11
ACVR1B	0.06	0.30
ACVR1C	0.04	0.05
ACVR2A	-0.25	-0.14
ACVR2B	-0.64	-0.78
ACVR2B-AS	0.18	0.18
ACVRL1	0.13	0.03
CDH2	0.55	-0.06
CFC1	-0.07	-0.06
CFC1B	0.03	-0.01
EOMES	-0.70	-0.76
FABP4	0.12	0.28
FOXF1	0.18	-0.04
GDF3	-0.98	-1.01
GSC	0.02	-0.08
GSC2	0.05	0.19
KDR	-1.24	-1.06
MESDC1	-0.03	-0.08
MIXL1	-0.35	-0.31
NCLN	-0.67	-0.78
NOMO1	-0.55	-0.37
NOMO2	-0.17	-0.14
NOMO3	-0.21	-0.33
SNAI1	0.02	0.11
SNAI3	-0.07	-0.16
T	-0.10	-0.12
TBX6	-0.04	-0.09
TWIST2	-0.09	0.29
WNT8A	0.00	-0.12

NEUROECTODERM		
symbol	day 5	day 10
FGF1	-0.05	-0.05
FGF2	-1.59	-2.48
FGF3	-0.05	-0.10
FGFR1	-0.63	-0.78
FGFR2	-0.18	-0.11
FGFR4	0.11	0.07
PARP1	-0.56	-1.47
PAX6	0.11	-0.14

PLURIPOTENCY		
symbol	day 5	day 10
ALPL	-2.29	-3.71
DNMT3B	-1.66	-3.14
LIN28A	-0.69	-2.23
LIN28B	-0.79	-1.32
nanog	-1.96	-2.25
POU5F1	-3.50	-5.15
SOX2	-2.53	-3.61
TRIM71	-2.09	-2.46

<u>Table S2. Related to Figure 2B:</u> Screen's hits associated with epithelial differentiation

symbol	gene	Involvement (references)
BMPR1B	Bone Morphogenetic Protein Receptor, Type IB	associated with epidermis differentiation (Botchkarev et al., 1999; Panchision & Pickel, 2001)
IKBKAP	Inhibitor Of Kappa Light Polypeptide Gene Enhancer In B-Cells, Kinase Complex-Associated Protein	promote epidermal differentiation (Hu et al., 2001)
IKBKE	Inhibitor of kappa light polypeptide gene enhancer in B-cells, kinase epsilon	
MAPK1	Mitogen-Activated Protein Kinase 1	viability and proliferation of keratinocyte (Scholl et al., 2007)
MAP2K1	Mitogen-Activated Protein Kinase Kinase 1	
MAP2K2	Mitogen-Activated Protein Kinase Kinase 2	
MAP2K4	Mitogen-Activated Protein Kinase Kinase 4	
MAP3K3	Mitogen-Activated Protein Kinase Kinase Kinase 3	
MAP3K12	Mitogen-Activated Protein Kinase Kinase Kinase 12	

Table S4. Related to Figure 2B: Screen's top candidate gene list

	GeneID	z-score
promoter candidates	STK33	4.04
	TEK	3.77
	MYLK	3.43
	GSK3B	3.38
	CDKN2B	3.3
	GCK	2.98
	MAP2K4	2.86
	MAPKAPK3	2.78
	PAK1	2.75
	CDKL1	2.72
	MYO3A	2.65
	SRPK2	2.64
	GTF2H1	2.62
	CDK11B,CDK11A	2.52
	MAP2K2	2.43
	MAP2K3	2.41
	TYK2	2.38
	STYK1	2.3
	RBKS	2.29
	CDK7	2.25
	GRK7	2.25
	**ADCK2**	**2.24**
	ADRB2	2.2
	ZAP70	2.18
	PHKA2	2.17
	CDKN3	2.16
	MVK	2.1
	TRAT1	2.06
	WNK4	2.04
	GK2	2.02
	GMFB	2.02
	XYLB	2
	IPPK	1.99
	JAK1	1.99
	CAMK2B	1.98
	PIP5K1A	1.97
	SOCS5	1.96
	DUSP10	1.92
	CDK5R1	1.9
	CSNK2B	1.89
	MAPK1	1.85
	SHPK	1.8
	IRAK3	1.77
	DGKQ	1.72
	DYRK2	1.72
	SCYL2	1.71
	NEK9	1.7

	GeneID	z-score
inhibitor candidates	CSNK1E	-1.55
	DBF4	-1.56
	ACVR2A	-1.57
	YWHAQ	-1.57
	CINP	-1.59
	MLKL	-1.59
	AATK	-1.6
	TRIB3	-1.61
	AAK1	-1.62
	PRKD1	-1.64
	**HIPK3**	**-1.66**
	EIF2AK3	-1.69
	EPHA3	-1.69
	FASTK	-1.7
	GUCY2F	-1.71
	POM121L10P,BCR	-1.71
	FGFR1	-1.72
	SRPK1	-1.72
	BMP2K	-1.73
	CHEK2	-1.73
	CHKA	-1.73
	EPHA4	-1.73
	EIF2AK1	-1.74
	TEX14	-1.74
	CHKB	-1.75
	BLK	-1.77
	**HIPK4**	**-1.77**
	TTBK2	-1.79
	TAF1L,TAF1	-1.83
	TPK1	-2.01
	SLK	-2.1
	TSSK1B,DGCR14,TSSK2	-2.14
	UCK2	-2.21
	TTK	-2.38
	PBK	-2.42
	TXK	-3.01

	CKS1B	1.69
	PAK2	1.69
	CARD10	1.68
	MST4	1.68
	TWF2	1.65
	AVPR1A	1.62
	CD4	1.59
	ILKAP	1.56
	TLR1	1.56
	ARAF	1.54
	CDK10	1.54
	PRKDC	1.52
	PRKCQ	1.51
	CD7	1.5
	ZAK	1.5

Table S5. Related to Figure 3D: Gene signature of endodermal, mesodermal, neuroectodermal tissue and of pluripotency.

ENDODERM		
**symbol**	**day 5KD**	**day 10KD**
KRT19	0.02	-1.93
CLDN6	-0.11	-1.80
SOX7	0.00	-1.59
CDH5	0.13	-1.54
CTNNBL1	-0.05	-1.13
KRT7	0.04	-0.99
CD14	-0.02	-0.51
SALL4	0.07	-0.47
FOXA1	-0.14	-0.46
GDF3	-0.02	-0.23
SOX17	0.14	-0.17
GATA6	0.04	-0.14
CDX2	0.03	-0.10
SOX1	0.01	-0.05
CXCR4	0.05	-0.04
HNF4A	-0.06	-0.02
MIXL1	0.17	-0.02
FABP1	-0.03	0.05
GATA4	0.08	0.06
VEGFA	0.06	0.07
FOXA2	-0.01	0.07
CD36	-0.07	0.15
PECAM1	-0.02	0.17
AFP	-0.27	0.17
HNF1B	0.03	0.18
UPK1B	0.06	0.28
EOMES	0.03	0.35
VEGFB	-0.05	0.47

MESODERM		
**symbol**	**day 5KD**	**day 10KD**
HAND1	-0.25	-3.08
WNT3A	0.27	-0.45
NOMO1	0.15	-0.44
ACVR1B	0.08	-0.40
TWIST1	0.02	-0.40
NCLN	0.14	-0.37
GDF3	-0.02	-0.23
TWIST2	0.08	-0.22
GSC2	0.02	-0.18
KDR	0.03	-0.17
ACVR2B-AS	0.01	-0.17
SNAI1	0.01	-0.09
SNAI2	0.02	-0.08
FOXF1	-0.09	-0.02
MIXL1	0.17	-0.02
SNAI3	-0.05	-0.01
GSC	-0.11	-0.01
NOMO2	0.02	-0.01
CFC1	0.03	0.02
CFC1B	0.04	0.02
TBX6	-0.10	0.07
T	0.06	0.07
ACVR1C	0.02	0.07
ACVR2B	-0.07	0.10
WNT8A	-0.02	0.10
MESDC1	-0.03	0.11
ACVRL1	-0.21	0.15
ACVR2A	0.08	0.22
EOMES	0.03	0.35
NOMO3	-0.05	0.43
ACVR1	0.02	0.74

NEUROECTODERM		
**symbol**	**day 5KD**	**day 10KD**
FGF1	0.01	0.01
FGF3	-0.03	0.11
FGFR1	-0.08	0.13
FGFR2	0.03	-0.17
FGFR3	-0.13	-0.91
FGFR4	0.01	-0.06
PARP1	0.04	-0.08
PAX6	-0.02	0.54

PLURIPOTENCY		
**symbol**	**day 5KD**	**day 10KD**
LIN28A	0.03	-2.87
DNMT3B	-0.01	-1.55
KLF4	0.06	-0.93
ABCG2	-0.21	-0.84
ACVR1B	-0.33	-0.81
TRIM71	0.17	-0.72
ALPL	0.15	-0.70
POU5F1	0.07	-0.42
TPBG	0.02	-0.15
LEFTY1	-0.05	-0.10
ACVR2B	-0.05	-0.06
FUT2	0.04	-0.04
LEFTY2	0.08	0.02
CFC1	0.03	0.02
TERT	0.11	0.04
LIN28B	-0.03	0.08
SOX2	0.03	0.54
KLF5	0.05	0.61
